# Synthesis of Fluorinated Nucleosides/Nucleotides and Their Antiviral Properties

**DOI:** 10.3390/molecules29102390

**Published:** 2024-05-19

**Authors:** Yugandhar Kothapalli, Ransom A. Jones, Chung K. Chu, Uma S. Singh

**Affiliations:** Department of Pharmaceutical and Biomedical Sciences, College of Pharmacy, University of Georgia, Athens, GA 30602, USA; yugandhar.kothapalli@uga.edu (Y.K.); ransom.jones@uga.edu (R.A.J.)

**Keywords:** nucleosides, nucleotides, antiviral, fluoro-nucleosides/nucleotides, ribose sugar, carbocyclic ring, fluorinated sugar, clinical development, clinical trials

## Abstract

The FDA has approved several drugs based on the fluorinated nucleoside pharmacophore, and numerous drugs are currently in clinical trials. Fluorine-containing nucleos(t)ides offer significant antiviral and anticancer activity. The insertion of a fluorine atom, either in the base or sugar of nucleos(t)ides, alters its electronic and steric parameters and transforms the lipophilicity, pharmacodynamic, and pharmacokinetic properties of these moieties. The fluorine atom restricts the oxidative metabolism of drugs and provides enzymatic metabolic stability towards the glycosidic bond of the nucleos(t)ide. The incorporation of fluorine also demonstrates additional hydrogen bonding interactions in receptors with enhanced biological profiles. The present article discusses the synthetic methodology and antiviral activities of FDA-approved drugs and ongoing fluoro-containing nucleos(t)ide drug candidates in clinical trials.

## 1. Introduction

In the past several decades, many fluoro-containing nucleos(t)ide drugs have been approved both in antiviral and anticancer therapies [1]. Nucleos(t)ides are fundamental nucleic acid fragments and are essential molecules for all living systems, including for the synthesis of DNA and RNA [2]. These molecules also play a significant role as chemotherapeutic agents in treating cancer and viral infections via selectively targeting certain enzymes of cancer and enzymes necessary for viral replication [3,4]. In the late 1980s, the unprecedented condition of acquired immunodeficiency syndrome (AIDS) caused by the human immunodeficiency virus (HIV) accelerated the discovery and development of nucleoside molecules as antivirals and since then, several FDA-approved nucleoside drugs have emerged in clinical practice [5]. Recently, it has been made evident that nucleos(t)ide-based drugs have played a prominent role in the cure and eradication of the COVID-19 infection, and these molecules have contributed significant aid in helping to overcome the pandemic caused by severe acute respiratory syndrome coronavirus-2 (SARS-CoV-2) [6].

Medicinal chemists engage in the design and synthesis of modified nucleos(t)ides with an altered nucleobase or sugar to discover new nucleoside-based therapeutic agents. The altered nucleobase and sugar techniques increase the selectivity and efficacy of nucleos(t)ides against specific viral enzymes without causing toxicity to the host [3,7]. In the past two decades, much advancement has been made in the design and synthesis of modified nucleos(t)ide analogs, and these endeavors invented various new classes of nucleos(t)ide-based drugs [8]. The selective insertion of fluorine atoms in a naturally or biologically active moiety often reveals an enhanced biological profile of interest. The electronegativity of fluorine and its capacity to add an additional hydrogen bond in receptors has gained much attention in drug design. In the area of nucleos(t)ide drugs, the incorporation of a fluorine atom either in the base or sugar also demonstrates better antiviral and anticancer activities and several fluoro-nucleos(t)ide drugs are in clinics. It has been estimated that more than 20% of drug candidates and 50% of agrochemicals contain one or more F atoms [9,10].

Fluorine is a magical atom in chemistry, which displays diverse pharmacological effects in biologically active molecules. The unique property of fluorine may substantially change the chemical, physical, and biological properties of active molecules [11]. It modulates and influences the pharmacokinetic and pharmacodynamic properties of drugs [12]. Incorporating a fluorine atom in organic moiety is beneficial due to its high electronegativity (3.98 on the Pauling electronegativity scale) [13]. The electronegativity of fluorine favors a firm and highly polarized C-F bond that improves the stability of small organic molecules. It also affects the acidic and basic properties of adjacent functional groups by impacting the overall pK_a_ of the molecule [14]. The greater stability of the C-F bond compared to the C-H bond can prevent oxidative metabolism, thus making the C-F bond more resistant to oxidation and degradation than the C-H bond and restricting the formation of undesired metabolites [15]. The relatively small size of fluorine (van der Waals radius of 1.47 Å) closely mimics hydrogen without changing the geometry of molecules and demonstrates additional hydrogen bonding to a receptor or enzyme with minimal steric effects [16]. Fluorine is a bioisostere of hydrogen that expresses better lipophilicity than hydrogen with a reduced basicity of organic molecules, which enhances the cell penetration of molecules and drives them for easy delivery to the active site [17]. It also generates a significant role in the oral bioavailability of drugs via better absorption. Furthermore, the mentioned rationales underscore the significance of fluorine insertion, elucidating its pivotal role in drug design and the discovery of novel clinical drug candidates [18].

The presence of a fluorine atom in the ribose or carbocyclic ring of nucleos(t)ide impacts the glycosidic bond strength and increases enzymatic and metabolic stability [19]. It also affects the dipole–dipole, gauche, and F-base interactions [15]. The potential activity and stability of fluorinated nucleos(t)ides varies according to the substitution of fluorine atom(s) in different positions on nucleoside [20]. It is predicated but not proven that fluorine at the 2′-*β*-position of the ribose favors a south conformation of the molecule and is linked to DNA virus activity. In contrast, the 2′-*α*-position favors a north conformation and demonstrates activity against RNA viruses [21]. The dynamic equilibrium between two furanose puckering forms is the characteristic 3′-*endo*/2′-*exo* or ‘*North*’ (*N*) and 2′-*endo*/3′-exo or ‘*South*’ (*S*) ring conformations [22].

The development of the fluorinated drug 5-fluorouracil (5-FU) as an anticancer agent prompted extensive interest in developing fluorinated nucleoside/nucleotide analogs (NA) as therapeutic agents [23]. This has further been accelerated by the rapid development of synthetic methodologies in organofluorine chemistry. The substitution of the hydroxy or hydrogen in the sugar part or base of nucleosides by the fluorine atom leads to minimal steric effects and enhances the metabolic stability of nucleos(t)ides [18]. The present review article sheds light on the synthesis and antiviral activity of fluorinated nucleos(t)ides. In this article, we emphasize the synthesis and therapeutic importance of fluorinated nucleos(t)ides as antiviral drugs, along with candidates that are currently under clinical trials.

## 2. Synthesis of Fluorinated-Nucleos(t)ides

Two main approaches have been implemented in the synthesis of fluorinated nucleos(t)ides: one is the direct fluorination of the nucleoside moiety (divergent approach), and the second is a coupling of a fluorinated sugar or nucleobase with each other (convergent approach) [21]. Direct fluorination on the nucleoside is a linear strategy that retains the original configuration of the nucleoside. However, the coupling of nucleobases or heterocycles with a ribose sugar or carbocyclic ring limits the construction of the selective desired *β*-conformation of the nucleoside and often deals with a poor stereoselective *N*-glycosylation or coupling [19].

## 3. General Synthetic Approaches for The Fluoro-containing Nucleosides

The *N*-glycosylation of the fluoro-containing sugar or carbocyclic ring system with nucleobases is a common strategy. The coupling of the fluorinated sugar with base or heterocycles is a preferable method, where the direct fluorination on nucleoside is either not feasible or retains low yields with fluorinating agents. There are several known modifications on ribose and carbocyclic rings at 1′,2′,3′,4′ and 5′ that have been reported [3,7]. Among these, 2-deoxy-2′-F-ribo nucleos(t)ides demonstrated potential antiviral activities. It is well known that the substitution of 2′-OH by fluorine slows or even abolishes the enzymatic catalysis of the glycosidic bond without changing the nucleoside’s original confirmation [24,25].

**Figure 1:**
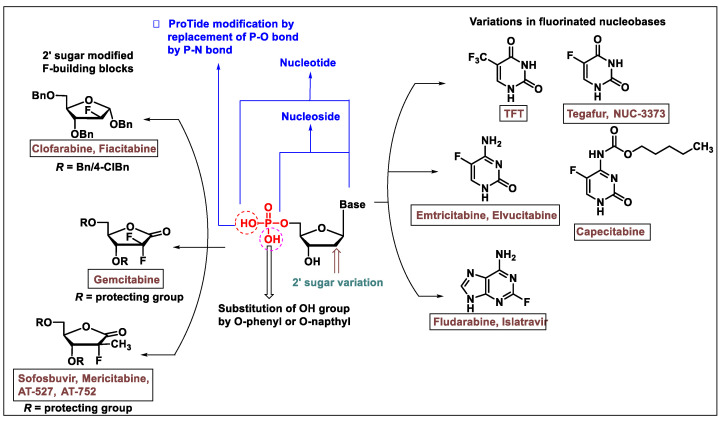
A representation of the general modifications at base, sugar, and prodrug forms of the approved nucleosides or nucleotide molecules, which are either approved drug or in various stages of clinical development.

The biological profile of *C*-2′-transformed nucleosides demonstrates enhanced antiviral properties of this class of molecules. Initially, Fox and co-workers introduced a series of 2′-deoxy-2′-fluoro analogs of uridine, 5-fluorouridine, and cytidine by treatment of 2,2′-anhydro nucleosides with hydrogen fluoride [26]. Since then, many strategies have been invented for the synthesis of 2′-fluoro nucleoside analogs, and these molecules have been examined thoroughly for biological properties (especially as an antiviral and anticancer agent). A general strategy for synthesizing fluoro-containing nucleos(t)ide is depicted in Figure 1.

The synthesis of the 2′-deoxy-2′-*β*-fluoro ribanofuranose (ara-F nucleosides) and 2-deoxy-2′-*α*-fluoro of nucleos(t)ide analogs are well-explored in the antiviral drug discovery. The insertion of fluorine at the 2′-position of ribose or carbocyclic rings enhances the antiviral activity. The convergent approach is more frequently used in the synthesis of 2′-*α*/*β*-fluoro nucleosides. Convergent approaches allow nucleoside chemists to perform variations in the sugar or carbocyclic moieties in diverse ways. For specific nucleos(t)ides where a fluorine atom is strategically placed at either purine or pyrimidine bases (as shown in Figure 1) the desired fluorinated bases are employed in *N*-glycosylation reactions to synthesize the required fluorinated nucleosides. Various drugs have been introduced that contain fluorine atoms on the nucleobase and are synthesized via a convergent approach [1,3,19].

## 4. Biological Importance of Fluorinated Nucleos(t)ide

In the past 50 years, based on fluorinated nucleos(t)ide molecules, several clinical candidates have emerged for the treatment of viral infections and cancers [1,27]. Majorly, fluoro-nucleos(t)ides and fluoro heterocyclic bases target thymidylate synthase (TS), ribonucleotide diphosphate reductase (RDPR) [28], and viral polymerases by which these molecules express anticancer and antiviral potency. Installing fluorine in the nucleos(t)ides also enhances the selectivity and specificity of these moieties towards the viral DNA and RNA polymerase [29].

## 5. Synthesis and Antiviral Activity of Fluorinated Nucleos(t)ides

In the past two decades, much progress has been achieved in the synthesis and development of fluoro-nucleos(t)ides as antiviral agents [30]. Further, the outburst of emerging viruses has been witnessed in recent years, and a lack of effective antivirals was slated. SARS-CoV-1 and 2, Middle East Respiratory Syndrome coronavirus (MERS-CoV), respiratory synthetical virus (RSV), Ebola, Zika, dengue, etc., and the spreading of new strains of herpes and poxviruses have the potential for a pandemic and epidemic burst [31]. Viral infections may be classified into three main types of infection. The first category encompasses life-threatening chronic infections caused by the human immunodeficiency virus (HIV), hepatitis C virus (HCV), and hepatitis B virus (HBV). The second class consists of acute viral infections such as influenza, and these infections are primarily non-lethal and self-resolving. The third class includes viral infections that are non-lethal but cause much economic impact [32].

**Figure 2:**
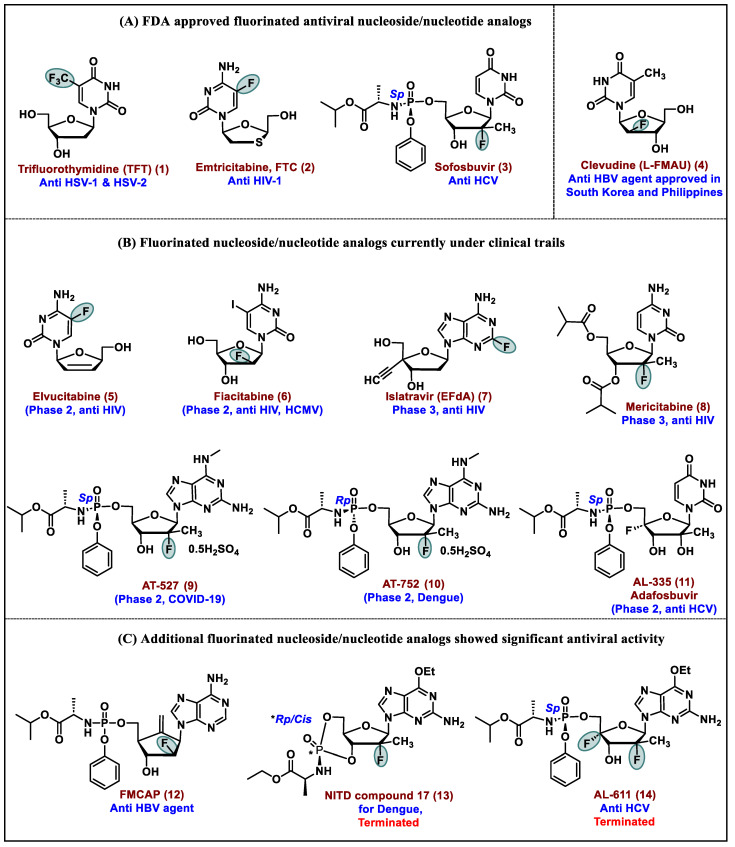
Chemical structures of the fluorinated antiviral nucleos(t)ides: (**A**) FDA approved antiviral drugs; (**B**) chemical structures of molecules under clinical trial; (**C**) chemical structure of preclinical molecule FMCAP (**12**) and terminated molecules **13** and **14**.

Nucleos(t)ides represent a special class of molecules for antiviral therapeutics. The viral polymerase enzyme is a well-established and historically explored target of the nucleos(t)ides [33]. The presence of fluorine in nucleos(t)ides makes them structurally unique in terms of various antiviral activities [34]. Mechanistically, nucleos(t)ide analogs inhibit the insertion of natural nucleos(t)ides either by competing with them or by inhibiting the viral polymerase enzymes. In general, nucleosides convert into the 5′-triphosphate form to exert biological activity. The viral polymerase catalytic residue expresses an essential role in the interaction with the primer and in the insertion of incoming 5′-phosphate nucleotides for chain elongation [35]. In the effort of viral inhibition, nucleosides demonstrate a two-way mechanism; they inhibit either the activity of viral polymerase or terminate the chain elongation of growing DNA or RNA strands and, in some cases, both. Based on the structural modification, several clinical candidates have been invented. Fluorinated antiviral nucleos(t)ide analogs and derivatives which are approved by the FDA, candidates under clinical trials, and molecules that exhibited potent antiviral activity are depicted in Figure 2.

### 5.1. Trifluorothymidine (TFT, 1)


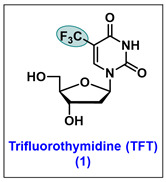


5-(trifluoromethyl)-*β*-D-2′-deoxyuridine, [CF_3_dUrd, (TFT)], known as trifluridine (Viroptic TM), is an antiherpetic drug approved by the FDA in 1980. TFT is used to treat eye infections caused by herpesviruses [36,37] and also exhibits antitumor activities [38,39]. Mechanistically, TFT converts into TFT-MP (monophosphate) by the thymidine kinase enzyme, which on further phosphorylation by cellular kinases, converts into active TFT-TP (triphosphate). After that, TFT-TP incorporate into viral DNA and inhibits viral DNA synthesis [40]. TFT also exhibits potent antiviral activity against herpes simplex virus (HSV-1 and 2), and it is prescribed to treat herpes simplex keratitis, keratoconjunctivitis, and other herpetic eye infections [41]. Additionally, the combination of trifluorothymidne/tipiracil is used for the treatment of metastatic colorectal cancer [42,43,44].

**Scheme 1:**
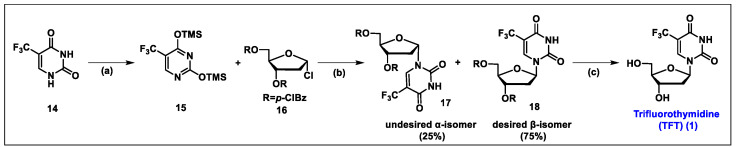
Synthesis of trifluorothymidine (TFT): (**a**) HMDS, 1,2-dichloroethane, TMS-Cl; (**b**) ZnCl_2_, dry CHCl_3_; (**c**) NaOMe in MeOH.

Initially, for the synthesis of TFT, several conventional methods were reported in the literature, such as enzymatic nucleic base transfer [45], the late-stage installation of trifluoromethylation on the nucleobase of 2′-deoxy-5-halouridine, and deoxyribose coupling with a silylated trifluoromethyl base [45,46]. These methods were not efficient for large-scale synthesis. Therefore, Kawakami et al. [47] reported a stereoselective glycosylation of chloro-sugar **16** with silylated 5-trifluoromethyl uracil trifluorothymine **15** in the presence of zinc chloride (ZnCl_2_, Scheme 1). This procedure has drawn great attention in synthesis to obtain the major desired *β*-isomer (**18**, in 75% yield) and minor undesired α-isomer (**17**, in 25% yield). The deprotection of the *p*-Cl benzoyl group of **18** in basic sodium methoxide conditions followed by the crystallization of crude in absolute ethanol affords TFT **1** in good yields.

**Scheme 2:**
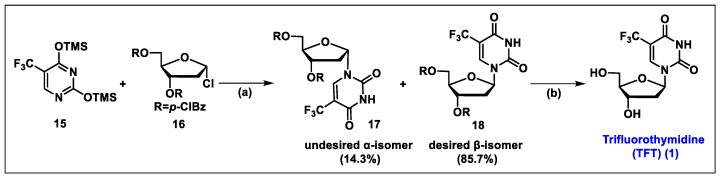
Improved synthesis of TFT via chloro intermediate **16**. (**a**) anisole 50 °C for 3.5 h; (**b**) 28% NaOMe in MeOH.

Later, Komatsu et al. [48] reported the synthesis of TFT via green glycosylation to improve the high stereoselectivity of desired *β*-isomer (18). In this reported method, an equimolar quantity of Cl sugar **16** was coupled with silylated trifluorothymine **15** in the presence of anisole (Scheme 2), which yielded a high *β*-selective coupled product **18**, compared to *α*-selective compound **17** in a ratio of 85.7: 14.3 in 71% yield.

The deprotection of the *p*-Cl-benzoyl group with a mixture of **17** and **18** was carried out with 28% NaOMe in MeOH followed by a neutralization of the reaction mixture by methanolic HCl solution, which affords a solid precipitate. The crude after treatment with butyl acetate (AcOBu) yields chiral pure TFT **1** (99.92:0.08 ratio of *β*:*α*) in 97% of good yield, and this process is in practice for the scalable synthesis of TFT.

In the in vitro antiviral evaluation, TFT exhibited potent activity against HSV-1 and -2 [49]. This drug presents some of the earliest developments of compounds against HSV infection. The administration of TFT is limited due to its cellular toxicity [50]. TFT is prescribed for the topical treatment of primary keratoconjunctivitis and recurrent epithelial keratitis caused by HSV-1 and HSV-2 [36]. It has also been used as a topical treatment for acyclovir-resistant chronic mucocutaneous genital HSV infections in HIV-infected patients [51].

### 5.2. Emtricitabine (FTC, 2)


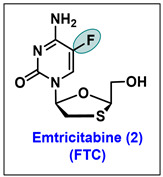


Emtricitabine is a synthetic (-) enantiomer of 2′,3′-dideoxy-5-fluoro-3′-thiacytidine (FTC, **2**). FTC is one of the most potent drugs for the treatment of human immunodeficiency virus (HIV) and has been licensed under the name of Emtriva™ and, in combination with tenofovir, known as Truvada™ [52]. FTC exhibited potent antiviral activity against HIV-1 and HIV-2 [53]. It is prescribed individually or in combination with other antiretroviral drugs. FTC is a chiral pure modified nucleoside reverse transcriptase inhibitor (NRTIs), in which a sulfur atom replaces the methylene group at the 3′ position of the 2′,3′-dideoxynucleoside substrate [54]. FTC has two chiral centers, with the *β*-L-configuration (*2R*,*5S*) being more potent (EC_50_ = 0.009 µM) and having greater metabolic stability and antiviral activity than its *β*-D-counter parts (EC_50_ = 0.84 µM) [55,56,57].

FTC is a 5-fluorinated analog of lamivudine (3-TC) and shares a common synthetic route with lamivudine (3-TC) for development [58]. Both are lifesaving drugs and are in high demand, for which several efficient synthetic routes were initially developed but were discontinued due to either low synthetic yield or tedious synthesis [54,59,60,61,62,63]. 

**Scheme 3:**
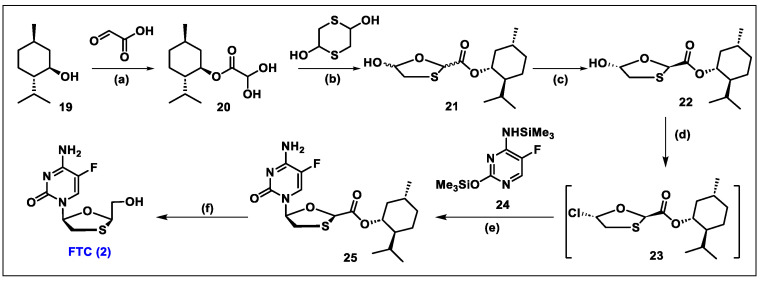
Synthesis of FTC via L-menthol 19. (**a**) 50% *w*/*v* glyoxylic acid, *p*-TSA, cyclohexane; (**b**) toluene, AcOH (cat.); (**c**) n-Hexane, NEt_3_ (cat.), crystallization; (**d**) SOCl_2_, DMF, DCM; (**e**) (i) NEt_3_, toluene, (ii) NEt_3_, H_2_O, n-hexane; (**f**) NaBH_4_, EtOH.

Later, the synthesis of chiral pure oxathiolane ring **23** was developed (Scheme 3), which, on selective glycosylation with the silylated 5-fluorocytosine, furnishes FTC in large quantities. Whitehead et al. developed an industrial production of the optically pure hydroxyoxathiolane ring **22**, (Scheme 3), which served as an essential key intermediate for the large-scale production of FTC. In the reported synthesis, L-menthol 19 serves as a chiral auxiliary, which assists in stereochemical outcome. L-Menthol 19 was treated with glyoxylic acid to render L-menthyl glyoxylate hydrate 20, which, on further reaction with dithiane diol, gave compound 21 as a mixture of four diastereomers. The initial crystallization of 21 in n-hexane with catalytic triethylamine (Et_3_N) afforded the desired diastereomer 22. 5-hydroxy-oxathiolane 22 was treated with SOCl_2_, in the presence of cat. DMF to give the reactive chloro intermediate 23. The coupling of pre-silylated fluorocytosine 24 with chloro intermediate 23 constructs glycosylated product 25 with a high stereoselectivity in *β* (desired)/*α* (undesired) isomeric ratio of 10:1. Further, the isomeric purity of **25** was enriched by solvent treatment. The 5′-ester reduction in **25** was carried out with NaBH_4_ in EtOH to give FTC (**2**) [64,65].

**Scheme 4:**
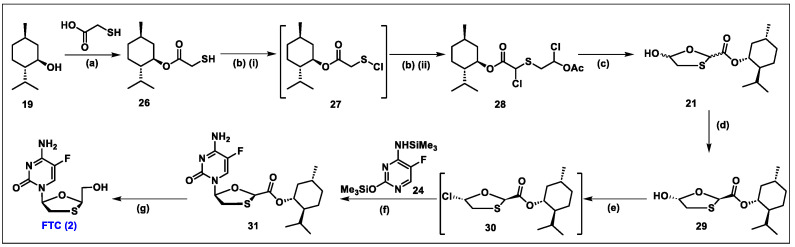
Improved synthesis of FTC, **2**. (**a**) thioglycolic acid, *p*-TSA·H_2_O, toluene; (**b**) (**i**) SO_2_Cl_2_, toluene; (**ii**) vinyl acetate; (**c**) H_2_O, CH_3_CN, 1M NaHCO_3_; (**d**) 1% NEt_3_ in hexanes; (**e**) MsCl, DMF, SOCl_2_; (**f**) HMDS, MsOH, DCM; (**g**) NaBH_4_, KH_2_PO_4_, MeOH-H_2_O.

Recently, Kashinath et al. reported a more robust and novel synthetic protocol with readily available, inexpensive starting materials (Scheme 4) [66]. The esterification of thioglycolic acid with L-menthol **19** gave intermediate **26**, which, via in situ treatment with sulfuryl chloride, afforded sulfenyl chloride intermediate **27**, which on further treatment with vinyl acetate gave **28**. Compound **28** was treated with H_2_O/CH_3_CN to give key intermediate **21**. The crystallization of **21** with 1% NEt_3_/hexanes gave the chiral pure compound **29**, which was further converted to the reactive chloro intermediate **30** through treatment with MsCl/SOCl_2_.

Intermediate **30** was condensed with silylated 5-fluorocytosine (**24**) to give coupled product **31**. After that, the reduction in **31** with NaBH_4_ furnished the final molecule FTC **2** [66]. Over the last three decades, significant contributions from various groups have led innovative synthetic methods like the crystallization of the diastereomeric mixture of **21** to produce the selective desired isomers **29**, and stereoselective *β*-*N*-glycosylation with chiral pure oxathiolane **29** has accelerated the scalable synthesis of FTC.

FTC(emtricitabine) is approved to treat HIV patients, with a 200 mg/day dosage in adults [67]. The in vitro evaluation of FTC against HIV in the lymphoblastoid, MAGI-CCR5, and peripheral blood mononuclear line expressed EC_50_ values of 0.64 μM to 1.3 nM, ranging from sub micromolar to nanomolar levels [67]. Also, in rats, no toxicity was observed in concentrations up to 31 times compared to normal human doses [68]. 

The preclinical toxicological assessment of FTC was extensively conducted and was very favorable for its clinical development. Taking the lead from the preliminary antiviral activities of FTC, various studies have been conducted to determine its potency in human lymphoblastoid T-cell lines (MT-4, CEM, or HT4-6C) acutely infected with a standardized infectious dose of a laboratory-adapted strain of HIV-1 (HIV-1_IIIB_ or HIV-1_LAV_) or HIV-2 (HIV-2_ZY_).

In these cell lines against laboratory-adapted strains of HIV-1, FTC demonstrated EC_50_ ranges of 0.009–1.5 µM in comparison to zidovudine (0.005–0.06 µM), lamivudine (0.07–9.8 µM), didanosine (8.5–16.0 µM) and zalcitabine (0.03–0.05 µM, Table 1) [69]. FTC expressed up to 10-fold enhanced activity than lamivudine against all viruses tested in all T-cell lines. FTC also exhibited synergistic antiviral effects in combination with other NRTI (abacavir, lamivudine, stavudine, tenofovir, zalcitabine, and zidovudine) and with non-nucleoside reverse transcriptase inhibitors (NNRTIs) like delavirdine, efavirenz, and nevirapine [52]. FTC, in combination with elvitegravir/cobicistat/tenofovir disoproxil fumarate as a single-tablet regimen (Stribild^®^), [manufacturer Gilead Sciences, Inc., Fosters City, CA, USA] is being prescribed for managing HIV infection in adults [70]. 

### 5.3. Sofosbuvir (3)


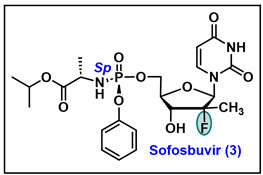


Sofosbuvir, also known as Sovaldi^®^, is a direct-acting antiviral (DAA) nucleotide drug that inhibits the NS5B polymerase of hepatitis C virus (HCV). Sofosbuvir, a phosphoramidate prodrug, is chemically named (*S*)-isopropyl 2-((*S*)-(((2*R*,3*R*,4*R*,5*R*)-5-(2,4-dioxo-3,4-dihydropyrimidin-1(2*H*)-yl)-4-fluoro-3-hydroxy-4-methyltetrahydrofuran-2-yl)methoxy)-(phenoxy) phosphorylamino)propanoate [71]. In this molecule, the 2′-position of the sugar is occupied by the *β*-methyl-α-fluoro group, which demonstrates interactions in the binding pocket of HCV-NS5B [72]. The published crystal structure of sofosbuvir diphosphate (PDB code 4WTG) revealed that the replacement of 2′-*α*-hydroxy of uracil with 2′-*α*-fluoro-*β*-methyl is beneficial. 2′-*α*-fluoro of sofosbuvir retains the hydrogen bonding with ASN 292 and disrupts the normal hydrogen bonding pattern that is usually expressed by the natural nucleotide substrates [73,74]. Sofosbuvir is a monophosphate prodrug that converts into its active uridine triphosphate in hepatocytes, exhibits chain termination during viral genome replication and hinders viral growth [75]. In combination with other drugs, sofosbuvir was approved by the USFDA to treat chronic HCV infection in adults and children ages ≥ 3 years [76]. The antiviral regimens, in combination with sofosbuvir, have revolutionized HCV management with a success rate of >90% and have provided a therapeutic tool to eradicate HCV infection. Sofosbuvir is administered orally, once daily, with no meal restriction. This drug also demonstrates good safety and efficacy with fewer side effects and exhibits minimal drug–drug interactions with pan-genotypic activity [77]. 

**Scheme 5:**
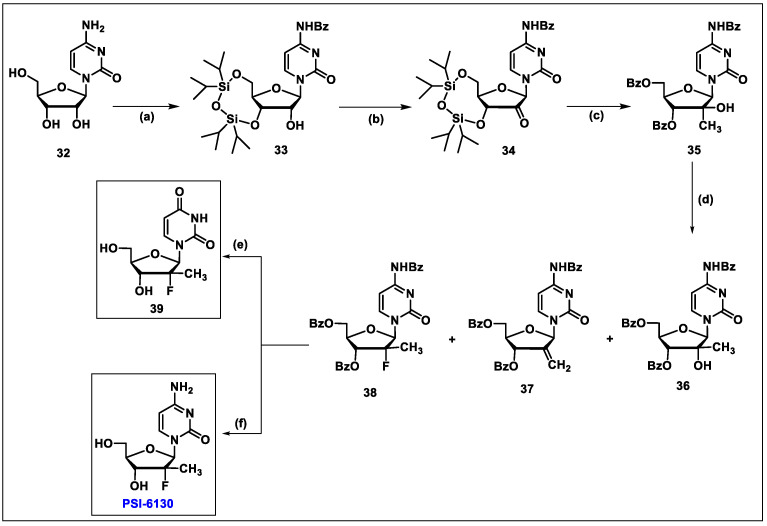
Synthesis of sofosbuvir precursor **PSI-6130** via cytidine **32**. (**a**) (i) benzoic anhydride, DMF; (ii) TIDPSCl_2_, DMF, (**b**) DMSO, TFAA, NEt_3_; (**c**) (i) MeLi, anhydrous Et_2_O (ii) 1M TBAF in THF, AcOH, THF; (iii) BzCl, anhydrous pyridine; (**d**) DAST, anhydrous toluene; (**e**) (i) 80% AcOH; (ii) NH_3_/MeOH; (**f**) methanol/NH_3_.

Synthesis of Sofosbuvir: In 2005, Clark et al. first reported the synthesis of pyrimidine nucleoside *β*-D-2′-deoxy-2′-fluoro-2′-*C*-methyluridine (Scheme 5) and its anti-HCV activity [78]. Cytidine **32** was reacted with benzoic anhydride in DMF, followed by the treatment with TIDPSCl_2_ to afford 3′ and 4′ cyclic protected compound **33**.

The oxidation of the 2′-alcohol of **33** with trifluoroacetic anhydride/DMSO under Swern oxidative condition gives 2′-ketone **34**. The methylation of the ketone with methyllithium at −78 °C exclusively affords 2′-*β*-methyl arabinoside intermediate. The deprotection of the 3′,5′-silyl groups with TBAF/AcOH, followed by benzoylation with BzCl/pyridine, produces 2′-*α*-hydroxy alcohol **35**. The nucleophilic fluorination of 2′-*α*-hydroxy of **35** with (diethylamino)sulfur trifluoride (DAST) gives the desired *β*-fluorinated intermediate **38** along with elimination and hydrolyzed byproducts **36** and **37** in low yields. To synthesize uridine analog **39**, compound **38** was refluxed in 80% AcOH, followed by debenzylation under basic conditions, which gave **39**. Similarly, the debenzylation of **38** yielded a cytidine analog (**PSI-6130**).

**Figure 3:**
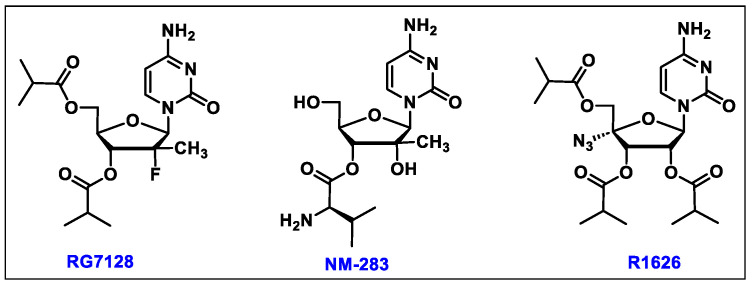
Modified 2′- cytosine analogs of **PSI-6130**.

Although in the earlier reported studies cytidine analog **PSI-6130** expressed potent activity against HCV, uridine analog compound **39** was found neither active nor cytotoxic [78]. Later, it was identified by Sofia et al. that due to the inadequate first step rate-limiting mono-phosphorylation of uridine, compound **39** was inactive [71]. However, in vivo metabolic studies indicate that cellular enzymes cytidine deaminase (CDA) convert **PSI-6130** to its uridine analog **39** [71]. Taking the lead from this finding, the phosphoramidate prodrug of uridine analog (**39**) was synthesized, which led to the invention of sofosbuvir **3**. Later, based on **PSI-6130**, several cytidine analogs (Figure 3) were developed as potential clinical candidates as anti HCV agents [71,79].

Initially, cytidine analog PSI-6130 with 2-*β*-methyl-*α*-fluoro substituents was synthesized via the synthetic route depicted in Scheme 5. However, after the discovery of sofosbuvir 3, this low-yielding process was incompatible for the large-scale synthesis of **PSI-6130**.

**Scheme 6:**
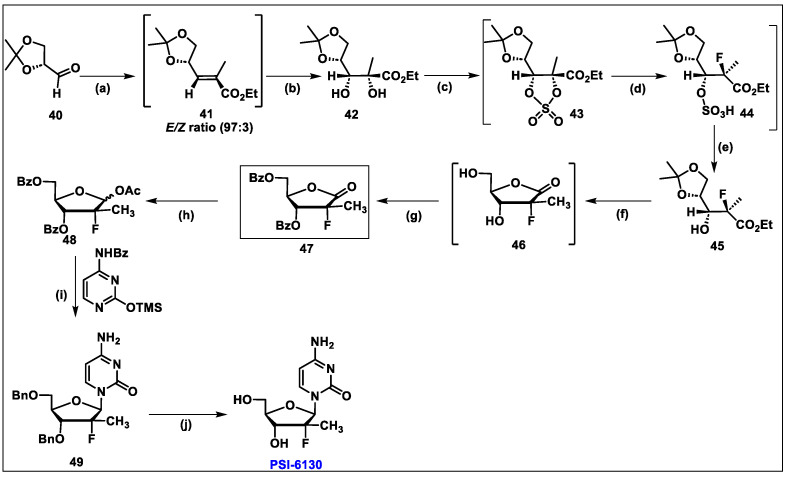
Scalable synthetic route of **PSI-6130**. (**a**) Ph_3_PC(Me)CO_2_Et, DCM; (**b**) KMnO_4_, acetone; (**c**) (i) SOCl_2_, NEt_3_, DCM; (ii) TEMPO, NaOCl; (**d**) tetraethylammonium fluoride hydrate, dioxane; (**e**) 2,2-dimethoxypropane, con. HCl; (**f**) Con. HCl, EtOH; (**g**) BzCl, pyridine; (**h**) (i) lithium tri-*tert*-butylaluminium hydride, dry THF; (ii) DMAP, Ac_2_O; (**i**) neat SnCl_4_; (**j**) 7M NH_3_/MeOH.

In 2009, Wang et al. published an efficient, scalable synthetic route for the preparation of the **PSI-6130** that is also being utilized for the extensive synthesis of sofosbuvir **3** [80]. To achieve large scale-synthesis, intermediate **47** was identified as a key scaffold (Scheme 6). The synthesis of **47** was commenced with commercially available 2,3-isopropylidene protected D-glyceraldehyde **40**, which, on reaction with (carbethoxyethylidene)triphenylmethyl phosphorane under Wittig conditions, gives the desired pentanoate ester **41** as an *E/Z* mixture in a ratio of 97:3. After that, intermediate **41** was treated with KMnO_4_ in acetone to construct *syn* diol **42**. Furthermore, **42** was treated with SOCl_2_ to form the cyclic sulfite, which on further oxidation with catalytic TEMPO/NaOCl renders cyclic sulfate **43**. The treatment of cyclic sulfate **43** with tetraethylammonium fluoride hydrate in dioxane gives a regiospecific *β*-fluorinated product **44** [81]. Compound **44** was treated with con. HCl in the presence of 2,2-dimethoxypropane in dioxane to give crude acyclic intermediate **45**, which on further treatment with con. HCl in EtOH at room temperature converts to unprotected lactone **46**. The diol protection of lactone **46** was carried out with benzoyl chloride in pyridine to afford fully protected key ribonolactone **47**. The reduction in the **47** with lithium tri *tert*-butoxyaluminium hydride gives an intermediate lactol, which in situ reacts with Ac_2_O in the presence of the DMAP of ribonolactol **48** in a ratio of 2:1. Next, the glycosylation of **48** with silylated *N*^4^ benzoylcytosine was performed with stannic chloride in chlorobenzene that exclusively gives the *β* form of protected nucleoside **49**. Finally, the debenzylation of **49** in methanolic ammonia affords **PSI-6130** in good yield.

**Figure 4:**
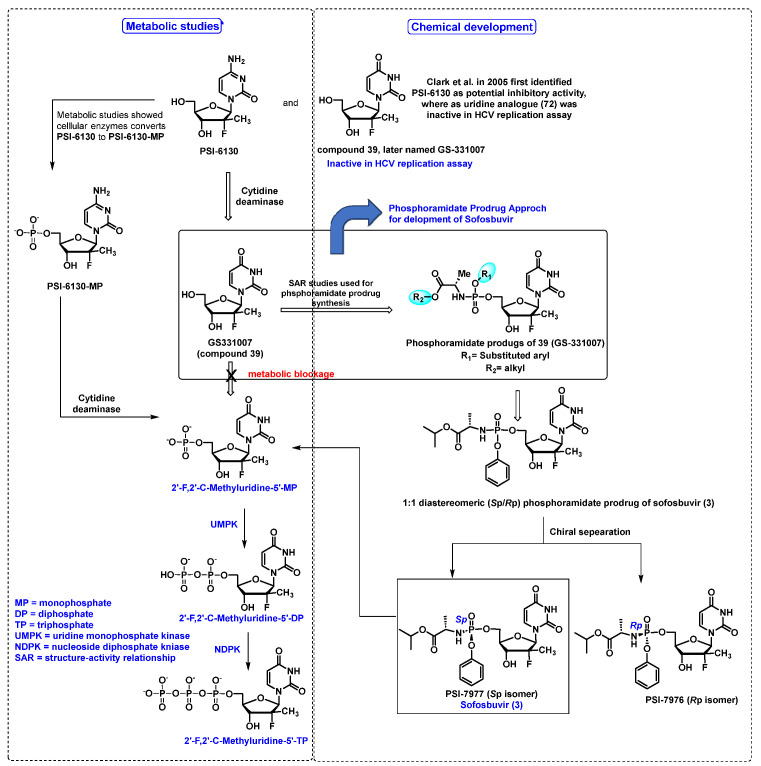
Metabolic pathway of PSI−6130 and subsequent phosphoramidate prodrug strategies for the development of sofosbuvir (**3**). (Clark et al. [79] in 2005).

Metabolic studies showed that **PSI-6130** triphosphate, an active metabolite in primary human hepatocytes, expresses a lower concentration and a shorter half-life. In preliminary in vitro studies, uridine nucleoside 39 in HCV replication assays was found inactive, whereas in later studies it was discovered that the triphosphate of 39 demonstrated the potent inhibition (Ki = 0.42 µM) of HCV NS5B [75,82,83]. Furthermore, it was evident that **PSI-6130** metabolizes to uridine 5′-triphosphate through cytidine-MP (monophosphate) via the cytidine deaminase enzyme (Figure 4). Due to the restricted first step rate-limiting monophosphorylation of compound **39**, it was unable to convert in its active form of triphosphate; for this reason, in initial studies, the core compound **39** was found inactive in HCV-cell replication assays, whereas the uridine triphosphate form of **39** reveals excellent potency with better stability and half-life in the intracellular system [71]. The metabolic conversion of **PSI-6130** and uridine analog **39** has been depicted in (Figure 4). To overcome the blockage of the first-step rate-limiting phosphorylation of **39**, phosphoramidate prodrug approaches were adopted. Phosphoramidate prodrug also caps the polarity of the free 5′-OH of nucleoside and enhances the lipophilicity of parental molecules that increase the cellular uptake of nucleotide. After penetration into the cell via facilitated passive diffusion, phosphoramidate prodrugs enzymatically cleaved off inside the cells to release the monophosphate of the parental molecule. Further sequential phosphorylation events lead to the formation of di or tri-phosphate derivatives, which subsequently generate the intended therapeutic effect [84,85].

Sofia M. J. et al. at Pharmasset have adopted phosphoramidate prodrug approaches for the development of sofosbuvir **3** and explored the complete structure–activity relationship (SAR) of these prodrugs on uridine nucleoside (**GS-331007** or compound **39**) [71]. 

**Scheme 7:**
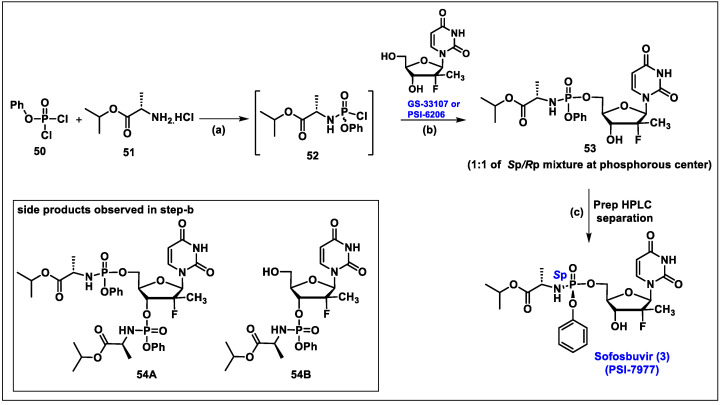
Synthesis of sofosbuvir (or phosphoramidate of GS-331007 or PSI-6206). (**a**) NEt_3_ or NMI, DCM; (**b**) NMI, THF; (**c**) prep HPLC purification.

**Scheme 8:**
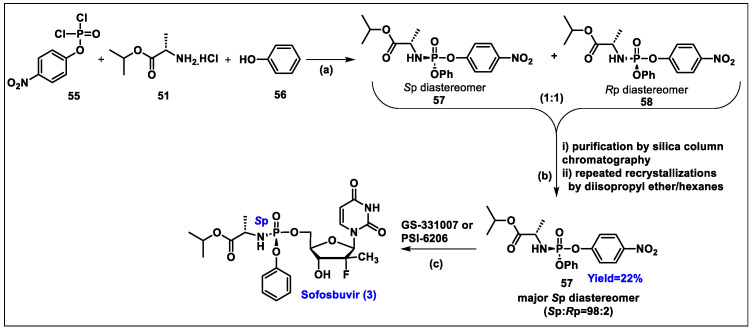
Synthesis of diastereomerically pure sofosbuvir (**3**) via intermediate **57**. (**a**) NEt_3_, DCM; (**b**) purification and recrystallization; (**c**) *t*-BuMgCl, THF.

Sofia et al. first described the diastereomeric separation of phosphoramidate prodrug isomers and reported that the in vitro *S*p isomer was 10-fold more potent than its *R*p isomer [71]. As shown in Scheme 7, the conventional synthesis of sofosbuvir faces several limitations regarding scalability and cost due to the chiral pure desired *S*p isomer isolates from a series of recrystallizations and chiral separation steps. Additionally, moisture-sensitive phosphorochloridate intermediate **52** poses rigor in handling and purification. To address these challenges, Ross et al. have developed a novel diastereomerically pure phosphoramidate synthesis of sofosbuvir **3** via coupling with the diastereomerically pure *S*p intermediate **57** in the presence of *tert*-BuMgCl as shown in Scheme 8 [86]. It is also noteworthy that various phosphoramidate prodrugs of PSI-6206 were synthesized with altered L-alanate esters in combination with various substituted phenoxy groups. However, the phenoxy substitute phosphoramidate prodrug with isopropyl-L-alanate ester demonstrated better anti-HCV activity, and later, it was named sofosbuvir (PSI−7977). 

**Scheme 9:**
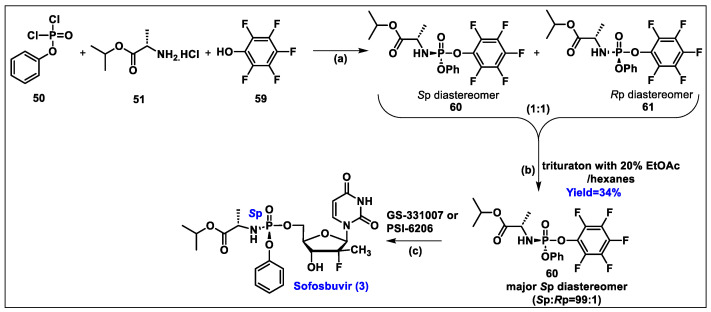
Synthesis of sofosbuvir via pentafluoro phosphoramidate reagent **60**. (**a**) NEt_3_, DCM; (**b**) purification and recrystallization; (**c**) *t*-BuMgCl, THF.

Commercially available *p*-nitrophenyl dichlorophosphate **55** was condensed with phenol **56** and L-alanine isopropyl ester hydrochloride **51** in the presence of triethylamine to give a 1:1 diastereomeric mixture of **57** and **58**. The diastereomeric mixture of **57** and **58** via repeated recrystallizations enriches single *S*p isomer **57** in 96% diastereomeric excess (*de*) in 22% yield. The **57** mixture, on coupling with uridine nucleoside **GS-331007** or **PSI-6206** in the presence of *tert*-BuMgCl in THF, affords sofosbuvir in a diastereomeric ratio of 98:2 (*S*p/*R*p). During these process developments, various phenolate leaving groups were evaluated; based on p*K*a values, 2,4-dinitrophenol-driven phosphoramidate reagents were found most reactive with nucleoside **GS-331007** or **PSI-6206** and lead to 5′ and 3′-disubstituted products. Eventually, it was determined that pentafluoro phenol-driven phosphoramidate reagent is the most optimum and promising for the synthesis of the desired *S*p isomer of sofosbuvir **3** with a greater than 98% diastereomeric excess (*de*) [86]. As depicted in Scheme 9, phenyl phosphorodichloridate **50** was reacted with L-alanine ester hydrochloride **51** in dry DCM and subsequently reacted with pentafluoro phenol **59**, resulting in a 1:1 crude mixture **60** and **61**. Trituration with 20% EtOAc/hexanes provides pure desired *S*p isomer **60** (99:1 ratio, *S*p/*R*p) in 34% yield, which on coupling with **GS-331007** or **PSI-6206** gives sofosbuvir **3** in good yield.

Currently, sofosbuvir is an FDA-approved oral drug for HCV treatment with an approved dose of 400mg/day in adults [87]. It is also used in combination with ledipasvir, known as Harvoni, and also prescribed in combination with daclatasvir [88].

Sofosbuvir demonstrated in vitro activity against all HCV genotypes. In HCV replicon assays, sofosbuvir showed EC_50_ values ranging from 0.014 μM to 0.11 μM for multiple full-length replicons across multiple genotypes (Table 2). During the outbreak of COVID-19, sofosbuvir was tested against SARS-CoV-2. However, it was found ineffective against this virus. Additionally, sofosbuvir has been tested against Zika virus in Huh7 cells, where it demonstrated an IC_50_ value of 4.1 µM and a CC_50_ value of >100 µM [89]; against the West Nile and dengue viruses in the Huh7 cell, sofosbuvir expressed an IC_50_ of 1.2 µM and EC_50_ of 4.9 µM, respectively (Table 2) [90,91]. 

### 5.4. Clevudine (CLV, 4)


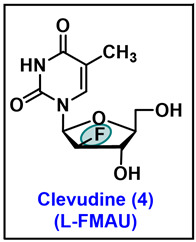


**Scheme 10:**
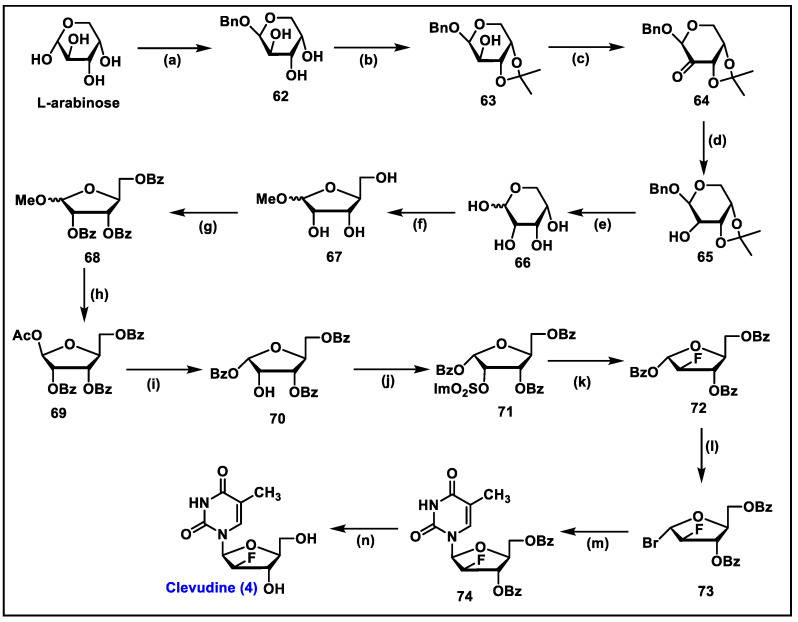
Synthesis of clevudine **4** from L-arabinose. (**a**) BnOH, HCl gas; (**b**) DMP, *p*-TsOH, acetone; (**c**) PDC, Ac_2_O, DCE; (**d**) NaBH_4_, MeOH; (**e**) 4% CF_3_CO_2_H; (**f**) 1% HCl in MeOH; (**g**) BzCl, pyridine; (**h**) Ac_2_O, AcOH, con, H_2_SO_4_; (**i**) (i) HCl (gas), dry DCM; (ii) H_2_O, acetonitrile; (**j**) (i) SOCl_2_, DCM, DMF; (ii) imidazole; (**k**) Et_3_N.3HF, EtOAc; (**l**) 45% HBr/AcOH; (**m**) silylated thymine, CHCl_3_; (**n**) NH_3_/MeOH.

Clevudine (CLV), chemically known as 2′-fluoro-5-methyl-β-L-arabinofuranosyluracil (L-FMAU), was initially synthesized in 1995 and has shown both anti-HBV and anti-Epstein–Barr virus (EBV) activity [92,93]. Clevudine is the unnatural L-analog of D-FMAU and is being marketed both in South Korea and the Philippines to treat hepatitis B virus (HBV) infection under the trade names Levovir™ and Revovir™. Chu et al. reported the first synthesis of it in 1995 involving a six-step initial synthesis from L-arabinose (Scheme 10). L-arabinose was benzylated with benzyl alcohol in HCl to give compound **62**, which was selectively protected with an isopropylidene group by treating with 2,2-dimethoxypropane (2,2-DMP) in the presence of catalytic *p*-TsOH to give **63**. The oxidation of the 2-hydroxy of **63** with PDC in dichloroethane (DCE), followed by the reduction with NaBH_4_, provides compound **65**. The debenzylation of **65** was performed with 4% TFA solution to render **66**, which on treatment with 1% HCl/MeOH solution via ring rearrangement furnishes the five-carbon ring sugar **67** (Scheme 10) [94]. 

Compound **67** was taken in pyridine and benzoylated with BzCl to give compound **68**, which was further treated with acetic acid and acetic anhydride with con. H_2_SO_4_ to give **69** (Scheme 10). Intermediate **69** was treated with saturated HCl gas in a solution of DCM followed by the hydrolysis and migration of benzoyl group from the carbon-2 to carbon-1 position to give tri benzoylated derivative **70**. To install the fluorine in the carbon-2 position, the hydroxy of **70** was converted to an imidazole sulfonate leaving group by treating **70** with thionyl chloride (SOCl_2_) in DMF and DCM, followed by imidazole to give **71**. The fluorination of **71** was carried out by triethylamine trihydrofluoride (Et_3_N.3HF) to afford compound **72**, which was further brominated with HBr/AcOH to obtain 1-bromo derivative **73**. The coupling of **73** with silylated thymine in chloroform yielded *β*-isomer **74** as a major compound with a trace of *α*-isomer, which was purified by the recrystallization in ethanol to give pure *β*-isomer **74** (Scheme 10) [92]. The final deprotection of benzoyl groups in ammonia methanol solution gave clevudine **4** in good yield. 

**Scheme 11:**
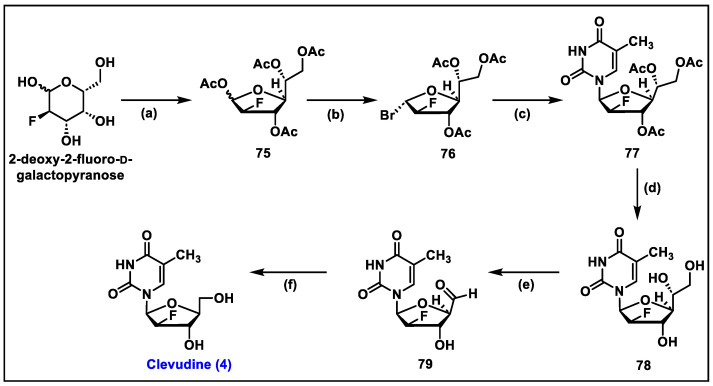
Synthesis of clevudine **4** from 2-deoxy-2-fluoro-D-galactopyranose. (**a**) Ac_2_O, pyridine; (**b**) 33% HBr/AcOH, DCM; (**c**) silylated thymine, CHCl_3_; (**d**) 7 N NH_3_/MeOH; (**e**) NaIO_4_, MeOH, H_2_O; (**f**) NaBH_4_, MeOH, H_2_O.

To develop a scalable synthetic route of clevudine **4**, a more robust and efficient synthesis was reported by several groups [95,96]. Tremblay et al. recently reported an efficient synthetic route with higher yields and fewer steps (Scheme 11) compared to the original synthesis to achieve this goal. Starting from the fluorinated galactopyranose, the isomerization of the pyranose was performed with acetic anhydride in pyridine to give **75**. The anomeric bromination of **75** with HBr in acetic acid in DCM, constructed bromo intermediate **76**, which on coupling with silylated thymine render coupled compound **77**. The deacetylation of the 5′ and 6′ hydroxyl with NH_3_ in methanol was performed, followed by the oxidative cleavage of diol with sodium periodate to produce compound **79**, which on reduction with sodium borohydride afforded clevudine **4** [96]. The reported synthesis in Scheme 11 represents a shortened six-step synthesis with the removal of the excessive protection of groups and is more efficient and safer for the multigram synthesis of clevudine **4**. 

Clevudine (L-FMAU), as mentioned, exhibited potent anti-HBV activity and is being prescribed for the treatment of chronic hepatitis B (CHB) infections. In early in vitro screening, clevudine has shown potent antiviral activity against multiple viruses. It expressed an EC_50_ value of 0.1 µM against HBV and an EC_50_ of 5.0 µM against EBV (Table 3) [92]. In P3HR1 cells infected with EBV, an EC_90_ of 5.0 µM was observed, with a CC_50_ of 1mM [97]. In HepAD38 cells infected with HBV, it showed an EC_50_ of 0.11 µM [98]. Clevudine has demonstrated effective HBV growth inhibition in various cells (Table 3). During the clinical trials in humans infected with CHB, median serum HBV DNA reductions of 4.49 and 4.45 log10 copies for 30 mg and 50 mg doses of clevudine were observed after 12 weeks compared to 0.20 log10 copies for placebo [99,100].

Furthermore, in a woodchuck HBV model, four weeks of clevudine therapy was well tolerated by chronically infected woodchucks, and clevudine showed an inhibition of woodchuck hepatitis virus (WHV) replication in a dose-dependent manner. Also, in this model, no toxicity was observed, and a significant antiviral effect was noted at a dosage of 0.1 mg/kg [101]. Clevudine also exhibited favorable antiviral effects in combination with other nucleoside antiviral drugs [98].

### 5.5. Elvucitabine (5)


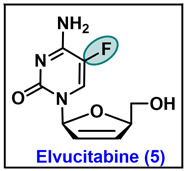


Elvucitabine, chemically known as L-*β*-2′,3′-didehydro-2′,3′-dideoxy-5-fluorocytidine (*β*-L-FD4C), is an L-cytosine nucleoside analog. Currently, elvucitabine is under investigation in phase 2 clinical trials (clinical trials.gov number: NCT00675844) [102]. It is a nucleoside reverse transcriptase inhibitor (NRTI), demonstrating potent antiviral activity against HIV. Elvucitabine intracellularly phosphorylates into its active 5′-triphosphate metabolite, which further inhibits the activity of HIV reverse transcriptase by competing with natural substrates, subsequently causing DNA chain termination after incorporation into viral DNA [103].

**Scheme 12:**
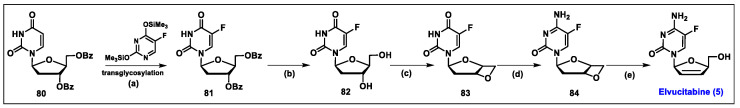
Synthesis of elvucitabine. (**a**) TMSOTf, CH_3_CN; (**b**) NH_3_/MeOH, sealed vessel; (**c**) pyridine, MsCl; (**d**) (i) 1,2,4-triazole, *p*-ClC_6_H_4_OPOCl_2_; (ii) NH_4_OH/dioxane; (**e**) ^t^BuOK, DMSO.

The initial synthesis of elvucitabine commenced with compound **80**. A trans-glycosylation of **80** with silylated 5-fluorouracil was carried out in the presence of TMSOTf as a catalyst to produce both *α* and *β* anomers in a 2:1 ratio (Scheme 12). The desired *β*-anomer of **81** was separated via silica gel column chromatography. After that, benzoyl groups were removed by treatment with the methanolic ammonia solution (NH_3_/MeOH) to afford 3′ and 5′ hydroxy intermediate **82,** which was treated with mesyl chloride to produce the cyclic ether **83**. Compound 83 was converted to cytosine analog 84 through the treatment of 83 with 4-chlorophenylphosphorodichloridate and 1,2,4-triazole in pyridine, followed by the addition of NH_4_OH in 1,4-dioxane. Finally, cyclic ether intermediate 84 was converted to the desired targeted elvucitabine 5 by treatment with potassium tert-butoxide in DMSO [104].

**Scheme 13:**
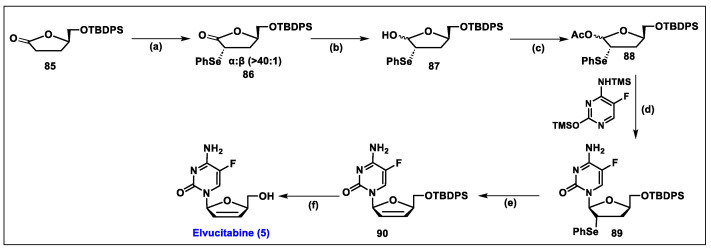
Stereoselective synthesis of elvucitabine from lactone **85**. (**a**) LiHMDS, THF then TMS-Cl, *N*-(phenylseleno)pthalimide, THF; (**b**) DIBAL-H, toluene; (**c**) Ac_2_O, NEt_3_, cat. DMAP, DCM; (**d**) TMSOTf dichloroethane; (**e**) 30% aq. H_2_O_2_, THF, then pyridine; (**f**) triethylamine trihydrofluoride.

Considering the impressive anti-HIV and anti-HBV activity of elvucitabine, a high-yielding and practical synthesis was needed. Chen et al. revisited the synthesis of elvucitabine and developed a robust, efficient, scalable, and stereoselective synthesis via lactone **85** (Scheme 13) [105]. A highly stereoselective phenylselenation of **85** was carried out with bulkier *N*-(phenylseleno)pthalimide to obtain carbon-2 phenylseleno intermediate **86** in a high selectivity (**84***α*/**84***β* > 40:1), affording the desired diastereomer **86***α* in good yield. To perform the *N*-glycosylation at *C*-1, first, the reduction in lactone **86***α* was accomplished with DIBAL-H in toluene to render lactol **87**. The acetylation of lactol **87** afforded the corresponding *C*-1 acetylated lactol **88** in qualitative yield. The coupling of **88** with the silylated fluoro cytosine in the presence of trimethylsilyl trifluoromethane sulfonate (TMSOTf) furnishes the desired cis nucleoside **89**. Furthermore, the treatment of **89** with hydrogen peroxide in the presence of pyridine constructs **90**, which, on desilylation with triethylamine trihydrofluoride, affords elvucitabine **5**. 

Antiviral evaluations showed that elvucitabine retains antiretroviral activity against NRTI-resistant viruses. Elvucitabine is generally well tolerated and after 10 days of monotherapy in 30 treatment-naive subjects with doses of 50, 100, or 200 mg q.d., a decrease in plasma HIV-1 RNA levels was reported [106]. This molecule is also in investigation in combination with other NRTIs for the cure of HIV infection [107]. In the preliminary in vitro evaluation, elvucitabine has demonstrated potent activity against both HIV and HBV. In CEM cell lines, it showed an EC_50_ value of 0.008 μM against HBV, while against HIV, it expressed an EC_50_ value of ~0.15 μM with a toxicity CC_50_ of 7 μM and SI values of 875 and 46, respectively [108]. In PBM cells, elvucitabine showed an EC_50_ value of 0.034 μM and CC_50_ value > 100 μM (Table 4) [109]. In the woodchuck model [woodchuck chronically infected with woodchuck hepatitis virus (WHV)], elvucitabine has demonstrated potent activity in suppressing HBV by inhibiting intrahepatic viral DNA synthesis [106].

### 5.6. Fiacitabine (6)


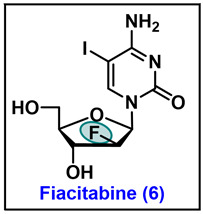


Fiacitabine (FIAC), 2′-deoxy-2′-fluoro-1-*β*-D-arabinofuranosyl-5-iodo-cytocine, is a pyrimidine nucleoside analog currently under a phase 2 study to treat cytomegalovirus (CMV) viremia infection in HIV infected patients [110]. Fox and his colleagues first reported the synthesis of fluoroiodoarabinosylcytosine or fiacitabine (FIAC) as an inhibitor of herpes simplex virus (HSV) and varicella-zoster virus (VZV) replication in the early 1980s, and the same group conducted phase 1 and phase 2 clinical trials. It demonstrates high in vitro activity against herpesvirus and hepadnaviruses and inhibits viral growth in its active triphosphate form. However, FIAC was not found to be a better analog compared to the currently used drugs acyclovir or zidovudine (AZT). Current clinical trials aim to develop FIAC in place of ganciclovir as an effective orally available therapeutic for HIV patients co-infected with CMV. The disadvantage associated with ganciclovir is that it is intravenously administered and associated with hematologic toxicity [110].

**Scheme 14:**
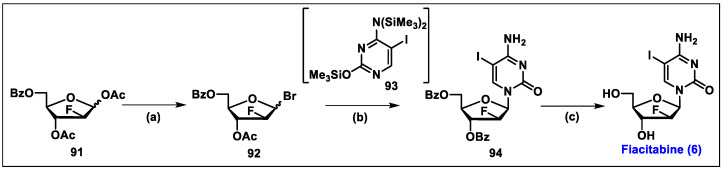
Synthesis of fiacitabine **6** via intermediate **92**. (**a**) dry DCM, HBr gas bubbling for 20 min at 0 °C then at 4 °C, 12h; (**b**) in situ added intermediate **93**; (**c**) NH_3_/MeOH.

The synthesis of fiacitabine **6** was carried out by acetyl-fluoro intermediate **91** (Scheme 14). The bromination of **91** with the HBr gas in DCM affords *C*-1 anomeric bromo compound **92**. The coupling of **92** with tris(trimethylsilyl)-5-iodo-cytosine (**93**) produces *β*-nucleoside **94**. The benzoyl deprotection of **94** was carried out by methanolic ammonia solution (NH_3_/MeOH) to give fiacitabine **6** as the final compound in 82% yield [111].

In initial in vitro screening, fiacitabine (FIAC) exhibited potent activity against HSV-1 and 2; however, later, it was found that it demonstrated antiviral activity against numerous other viruses. Preliminarily FIAC expressed an EC_50_ value of 0.01 µM against HSV 1 and 2, respectively, with ID_50_ value of 8.6 µM (Table 5). In in vitro assays in rabbit kidney_13_ (RK_13_) monolayers against equine rhinopneumonitis virus 1 (EHV-1), Aujeszky’s disease virus also known as Suid herpes virus 1 (SHV-1), and infectious bovine rhinotracheitis (BHV-1), fiacitabine showed an activity of IC_50_ values of 0.09–0.18 µM, 0.25–7.0 µM, and 0.1–3.0 µM, respectively [112]. Additionally, fiacitabine revealed an EC_50_ value of 0.4 µM against EBV in P3HR-1 cells and an EC_50_ of 1.1 µM against Kaposi’s sarcoma-associated herpesvirus (KSHV) in BCBL-1 cells, with CC_50_ values of 22 µM and 49 µM [113]. In vitro, FIAC proved to be very active against cytomegalovirus (CMV) ED_50_ of 0.6 µM, compared to brivudine and acyclovir [114].

In a double-blind clinical trial on VZV treated with fiacitabine at a dosage of 200 mg/m^2^ twice daily, fiacitabine expressed better antiviral potency compared to vidarabine (a now discontinued drug for the treatment of VZV infection) [115]. It was licensed to Bristol Myers Squibb (BMS) for further development as an antiviral agent to cure HIV patients coinfected with the CMV [110].

### 5.7. Islatravir (EFdA, 7)


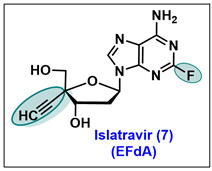


**Figure 5:**
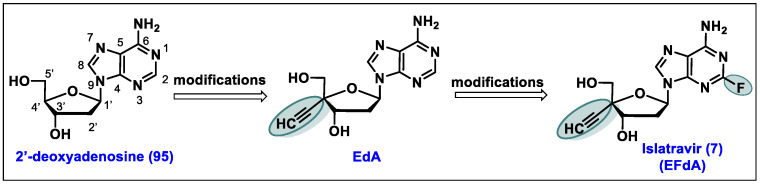
Chemical modification of 2′-deoxyadenosine (**95**) for the development of islatravir (EFdA).

Islatravir (ISL, MK-8591), 4′-ethynyl-2-fluoro-2′-deoxyadenosine (EFdA), is an investigational drug. Currently, it is under phase 3 clinical trials (clinical trials.gov number: NCT04233879) in the form of a fixed-dose combination (doravirine + islatravir) for the treatment and prevention of HIV-1 infection [116,117,118]. Islatravir is a nucleoside reverse transcriptase translocation inhibitor (NRTTI) that is different than other inhibitors of such class and inhibits HIV growth through multiple mechanisms. It is a modified analog of 2′-deoxyadenosine (**95**) at the 2- position of the base and 4′-position of sugar (Figure 5) and was first reported by Ohuri and co-workers [119,120].

During the early structure–activity relationship (SAR) studies, the lead compound 4′-ethynyl-2′-deoxyadenosine (EdA) exhibited excellent activity against HIV through the inhibition of HIV-1 reverse transcriptase. However, it was examined that EdA was susceptible to adenosine deaminase (ADA) degradation. To address this issue, a fluorine atom was inserted at the 2-position of the base; this change made 4′-ethynyl-2-fluoro-2′-deoxyadenosine (EFdA) as off-target for the ADA and expressed a prolonged intracellular half-life in its active triphosphate form [120]. The following exclusive key features make islatravir a unique clinical NRTTI.

- The 3′-OH group of EFdA resembles the natural substrate and readily inhibits viral polymerase.
- 2-F substitution on the adenine base imbues resistance towards oxidative adenosine deaminase (ADA) and contributes to its long half-life.
- The 4′-ethynyl group (4′-E) of EFdA is responsible for blocking the primer translocation and causes immediate chain termination, which mimics HIV reverse transcription [117,121,122].

**Scheme 15:**
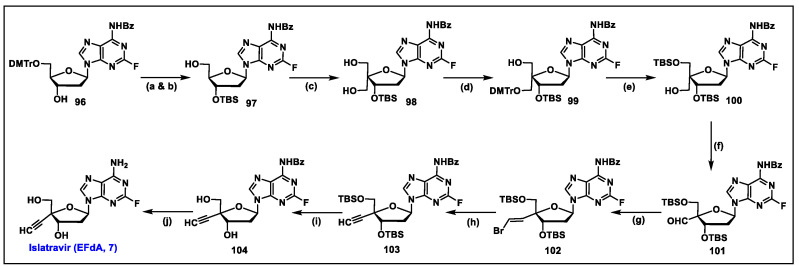
Synthesis of islatravir from the compound **96**. (**a**) TBS-Cl, imidazole, DMF; (**b**) TsOH.H_2_O, MeOH, CHCl_3_; (**c**) (i) EDC.HCl, pyridine, TFA, toluene, DMSO; (ii) aq. CH_2_O, 1*N* NaOH, dioxane; (iii) NaBH_4_, EtOH; (**d**) DMTrCl, NEt_3_, DCM; (**e**) (i) TBSCl, imidazole, DMF; (ii) TsOH.H_2_O, MeOH, CHCl_3_; (**f**) EDC.HCl, pyridine, TFA, toluene, DMSO; (**g**) PPh_3_=CHBr, THF; (**h**) *^t^*BuOK, THF; (**i**) TBAF, THF; (**j**) NH_4_OH, MeOH.

The synthesis of islatravir was originally reported by Ohuri et al. [123], and started from 2′-deoxyadenosine **96** via protection and deprotection methods to produce intermediate **97** (Scheme 15). Compound **97** was subjected to Moffatt oxidation to obtain 5′-carbaladehyde, which on aldol condensation gives 5′-diol **98**. The selective protection of the 5′-*α*-hydroxymethyl group of diols **98** was carried out with 4,4-dimethoxytrityl chloride in the presence of triethyl amine (Et_3_N) to afford the selective trityl-protected compound **99**. The protection of 5′-*β*-hydroxymethyl of 99 with TBDMS and the deprotection of DMTr furnishes the critical intermediate 100. The oxidation of the 5′-*α*-hydroxymethyl of 100 by Moffatt oxidation renders aldehyde 101, which on treatment with bromomethyltriphenylphosphonium bromide, furnishes bromo olefin compound 102. Furthermore, the dehydrobromination of 102 with *t*-BuOK in THF results in an alteration to the 4′-ethynyl 103 derivative. Finally, the sequential deprotection of TBS and benzoyl-protecting groups yielded 4′-ethynyl-2-fluoro-2′-deoxyadenosine (**EFdA**, 7). 

Due to the clinical importance of islatravir, several groups have developed linear synthetic routes for this clinical candidate via multiple protecting group manipulation approaches [124,125,126]. However, in these efforts, significant challenges were involved in inserting a stereo chemically pure ethynyl at the β-position of 4′-C, retaining the desired anomeric 1′-C configuration of islatravir. Recently, researchers at Merck & Co., Inc., Rahway, NJ, USA, using a bio-catalytic cascade method, have published two novel approaches for the large-scale synthesis of islatravir [127,128].

**Scheme 16:**
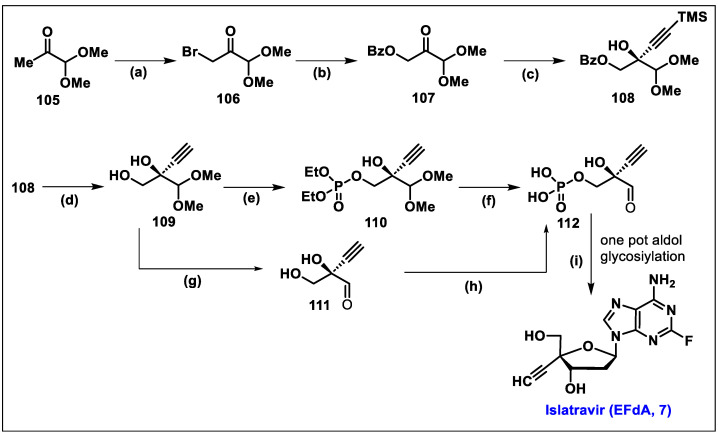
Synthesis of islatravir via a bio catalytic cascade method from compound **105**. (**a**) 5 mol% CaCl_2_, cyclohexylamine, then NBS, 16 h, rt, then IRA-120, 4 h; (**b**) KOBz, cat.Me_4_NBr, CH_3_CN; (**c**) ZnEt_2_, TMS acetylene, (1*R*,2*S*)-1-phenyl-2-(pyrrolidin-1-yl)propan-1-ol, toluene, THF (1:1); (**d**) NaOEt, EtOH; (**e**) diethyl phosphorochloridate, pyridine, DCM; (**f**) TMSBr, 2-methylbut-2-ene, DCM; (**g**) Amberlite IRA-120; (**h**) co-immobilized pantothenate kinase–acetate kinase, acetyl phosphate, ATP; (**i**) sucrose, acetaldehyde, 2-F-adenine, deoxyribose 5-phosphate aldolase (DERA), phosphopentomutase (PPM), purine nucleoside phosphorylase (PNP), sucrose phosphorylase (SP), Cat. MnCl_2_, PIPES/ TEOA in H_2_O, pH 7.6–7.5, 35 °C.

The synthesis began with pyruvic aldehyde dimethyl acetal **105**, which reacted with cyclohexylamine and calcium chloride in methyl *tert*-butyl ether (MTBE) to produce crude imine, which was treated with NBS in MTBE/hexanes to give brominated compound **106** (Scheme 16). The intermediate **106** was further reacted with potassium benzoate in the presence of cat. tetrabutylammonium bromide in acetonitrile to obtain benzoate protected diethyl acetal **107**. The enantioselective addition of TMS acetylene to the ketone of **107** was carried out in the presence of diethylzinc with respective (1*R*,2*S*)-1-phenyl-2-(pyrrolidin-1-yl)propan-1-ol ligand to afford highly enantioselective additive product **108** with a 95% *ee*.

The absolute stereochemistry of **108** was established by single X-ray diffraction. Intermediate **108** was subjected to one pot deprotection by using NaOEt to give diol **109**. The selective phosphorylation of the primary alcohol of **109** with diethyl phosphorochloridate afforded the key scaffold **110**, which, on the hydrolysis of phosphate esters with TMS-Br in the presence of 2-methyl-2-butene, rendered phosphoric acid **112**. Finally, the one-pot enzymatic aldol glycosylation of **112** with 2-F-adenine afforded islatravir in a 74% yield. Alternatively, intermediate 112 can also be synthesized by enzymatic phosphorylation by utilizing acetyl phosphate and adenosine triphosphate via intermediate 109, as shown in Scheme 16 [127].

Merck has initiated phase 3 clinical studies of oral islatravir in combination with doravirine to cure HIV-1 infection. Preliminarily, islatravir has shown excellent activity against both mutant and wild-type HIV. In in vitro studies against wild-type HIV, islatravir expressed an EC_50_ of 0.068 nM, while against mutant M184V, an IC_50_ of 3.1 nM was expressed; against multiple drug resistant (MDR) HIV, an IC_50_ of 0.15 nM was expressed (Table 6). Comparably, the cytotoxicity (CC_50_) of islatravir in the MT-4 cells was 7500 nM, with a very high selectivity index (SI) [123].

Furthermore, the anti-HIV activity of islatravir was evaluated in primary human cells and HIV-infected humanized mice. Similarly, it exhibited potent antiviral activity against HIV in phytohemagglutinin-stimulated peripheral blood mononuclear cells (PBMCs), and an IC_50_ value of 0.25 nM with a SI of 184,000 [129]. Islatravir also retained anti-HIV potency against 12 different HIV clinical isolates from multiple clades (A, B, C, D, and CRFF01_AE) [129]. Studies revealed that islatravir is quickly absorbed by oral administration and crosses the blood–brain barrier (BBB). Initial dosage indicated that the daily oral administration of islatravir at a low dose (1 to 10 mg/kg/Day) was highly effective in protecting humanized mice from the HIV infection, and within a week of therapy at a higher dose of 10 mg/Kg/day oral, islatravir completely suppressed HIV RNA to undetectable levels [129]. Currently, it is an investigational drug and is being developed in combination to prevent and treat HIV infections.

### 5.8. Mericitabine (8)


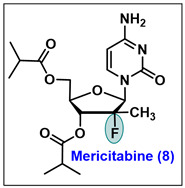


Mericitabine, [(2′*R*)-2′-deoxy-2′-fluoro-2′-methylcytidine 3′,5′-bis(2-methylpropanoate)], is a 3′,5′-diisobutyryl ester prodrug of the cytidine nucleoside analog that is directly prepared from compound **PSI-6130** [78]. This drug is orally administered and is a potent and selective inhibitor of HCV NS5B viral RNA dependent RNA polymerase (RdRp). After oral administration, mericitabine prodrug (RO4995855) is rapidly absorbed and converted to its parent molecule (*β*-D-2′-deoxy-2′-fluoro-2′-*C*-methylcytidine, PSI-6130) which is subsequently metabolized to its inactive uridine (RO5012433) metabolite. The parental molecule is absorbed by hepatocytes, where it undergoes phosphorylation to form cytidine monophosphate. It is then further converted into its active forms of cytidine triphosphate (RO4995855-tp) and uridine triphosphate (RO5012433-tp) [82,130,131,132]. 

In vitro studies have demonstrated that that two structurally distinct metabolites, cytidine triphosphate (RO4995855-tp) and uridine triphosphate (RO5012433-tp), exhibit selective, potent, and non-cytotoxic inhibitors of the viral HCV NS5B RdRp replication. This finding led to the discovery of sofosbuvir [71]. It was also speculated that mericitabine might express high potency in combination therapy. Currently, mericitabine is under phase 2 clinical trials (clinical trials.gov number: NCT01482403, NCT01482390) in combination with other drugs to treat chronic HCV infection [133].

**Scheme 17:**
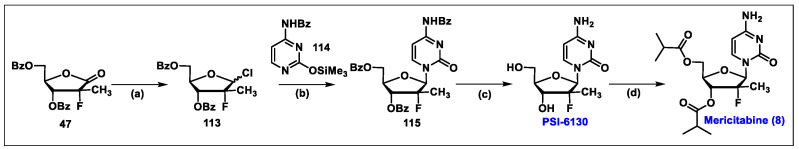
Large scale synthesis of mericitabine from intermediate **47**. (**a**) Red-Al, TBAB, SO_2_Cl_2_, toluene and butyl acetate; (**b**) SnCl_4_, DCM; after that aq. AcOH (93:7); (**c**) NaOMe (25% *w*/*w*) in MeOH; (**d**) isobutyryl chloride, NEt_3_, DMAP, THF.

In early 2013, a large-scale synthesis of mericitabine was reported [134]. The procedure entails the reduction of ribanolactone scaffold **47** (a common scaffold used in the synthesis of sofosbuvir as shown in Scheme 6). with Red-Al (Vitride®), in a mixture of toluene and butyl acetate. Subsequently, a catalytic amount of tetrabutylammonium bromide (TBAB) was added, and then treated with sulfuryl chloride to give chloride **113** as *α*/*β* anomeric mixture. Furthermore, the coupling of the silylated cytosine **114** with chloro intermediate **113** was carried out in the presence of tin (IV) chloride in DCM to afford the coupled product **115** as a major *β*-isomer (Scheme 17). The debenzylation of **115** with NaOMe in MeOH yielded the cytidine analog of **PSI-6130**, which on esterification with isobutyryl chloride gives targeted mericitabine, **8** [134]. 

In vivo, after absorption, mericitabine metabolizes into the parental molecule, PSI-6130. This molecule is an advanced nucleoside inhibitor of HCV RdRp with excellent oral bioavailability. In preliminary screenings, mericitabine and its active triphosphate have shown potent activity against HCV in a Huh7 replicon assay (IC_50_ 0.6 µM), along with activity against both native HCV replicase (IC_50_ 0.34 µM) and HCV recombinant RdRp cell-free enzyme assay (IC_50_ 0.13 µM, Table 7) [82,135]. 

**Figure 6:**
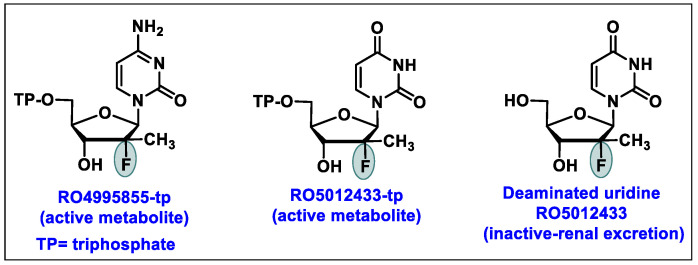
Active metabolic intermediates of mericitabine.

Mericitabine is active against all HCV genotypes but was most extensively examined against genotype 1. Its active triphosphate form (RO4995855-TP, Figure 6) and deaminated uracil triphosphate form (RO5012433-TP) have almost similar potency to mimic the RNA synthesis of HCV with an IC_50_ value of 0.34 and 1.19 µM, respectively. In in vitro studies in a transient replicon system, **PSI-6130** was equipotent against genotype 1a and 1b, with an EC_50_ ranging from 0.6 to 1.4 µM for various subtype 1b clinical isolates and 0.20 to 0.43 µM for the different subtype 1a isolates [136]. In a phase 1 monotherapy study conducted with 32 patients, mericitabine demonstrated a mean 2.7 log_10_ reduction in the viral RNA with a dose of 1500 mg BID [137]. However, its combination with interferon/ribavirin (IFN/RBV) achieved undetectable RNA levels, and in one of the phase 2b clinical trials, mericitabine in combination with IFN/RBA has demonstrated potency in the suppression of HCV RNA [138].

### 5.9. Bemnifosbuvir (AT-527, 9)

**Figure 7:**
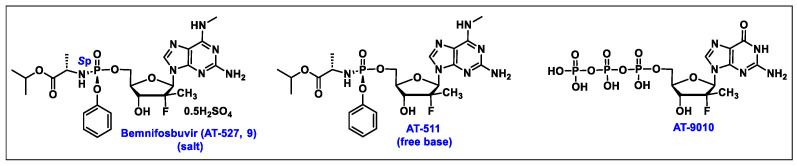
Structures of bemnifosbuvir (**AT-527**, **9**) and **AT-511**.

Bemnifosbuvir (**AT-527**, **9**) is an orally active double prodrug of the guanosine nucleotide analog of 2′-fluoro-2′-*C*-methylguanosine-5′-monophosphate that demonstrated potent in vitro and in vivo activity against HCV [139]. **AT-527** is a phosphoramidate prodrug in which the sugar moiety is identical to the clinically approved anti-HCV drug sofosbuvir. During the COVID-19 pandemic, it was found that the free base of **AT-527**, **AT-511** (Figure 7) expressed excellent antiviral activity against SARS-CoV-2 by selectively inhibiting the viral RdRp [140]. In in vitro evaluations, **AT-511** did not hinder human DNA polymerases or exhibit cytotoxicity, including mitochondrial toxicity at concentrations up to 100 μM. In the host cell, **AT-527** (*S*p isomer) metabolizes to its pharmacologically active triphosphate form (**AT-9010**, Figure 7), also known as 2′-fluoro-2′-*C*-methylguanosine-5′-triphosphate, which unveiled a dual mechanism of action including the chain termination of SARS-CoV-2 and the inhibition of the Nidovirus RdRp-associated nucleotidyl transferase (NiRAN) domain [140,141]. ATEA Pharmaceuticals is currently developing bemnifosbuvir (**AT-527**, **9**), and it has entered phase 3 clinical trials for the treatment of COVID-19 diseases and is currently in phase 2 clinical trials for HCV infection as a combination therapy (AT-527+ Ruzasvir) [142].

**Scheme 18:**
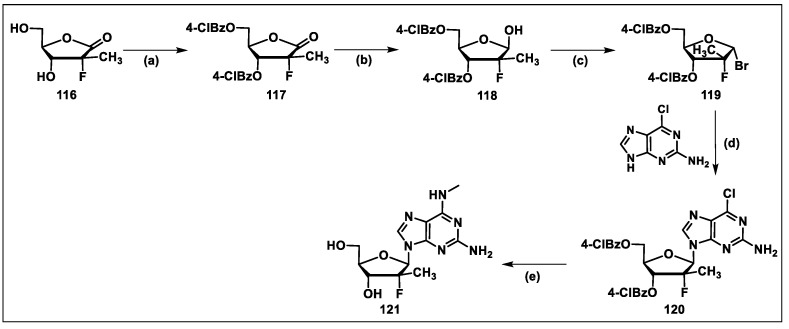
Synthesis of bemnifosbuvir key nucleoside **121** from lactone **116**. (**a**) 4-Cl-benzoyl chloride, NEt_3_, THF; (**b**) LiAl(OBu^t^)_3_H, THF; (**c**) TPP, CBr_4_, DCM; (**d**) KOBu^t^, ^t^BuOH, CH_3_CN; (**e**) 28% aq. MeNH_2_, THF.

The scalable synthesis of a critical nucleoside of bemnifosbuvir, **121,** is depicted in Scheme 18 [143]. The synthesis commenced with the protection of 3- and 5-hydroxyl groups of lactone **116**; it was treated with 4-Cl benzoyl chloride in pyridine to give protected lactone **117**. Fully protected lactone **117** was subjected to reduction with a bulkier reducing reagent tri-*tert*-butoxyaluminium hydride to afford lactol **118**, which on crystallization in MeOH/H_2_O renders 1-*β*-lactol with >95% purity exclusively. The bromination of **118** with CBr_4_ in the presence of triphenylphosphine in DCM yielded 1-*α*-bromo ribofuranose **119**. After that, the coupling of 6-Cl purine with **119** was carried out via S_N_2 glycosylation under basic potassium *tert*-butoxide condition to produce coupled product **120**. The *N*-methylation at the *C*-6 position of the base was achieved by treating intermediate **120** with 28% MeNH_2_ to afford the final targeted nucleoside **121**.

**Scheme 19:**
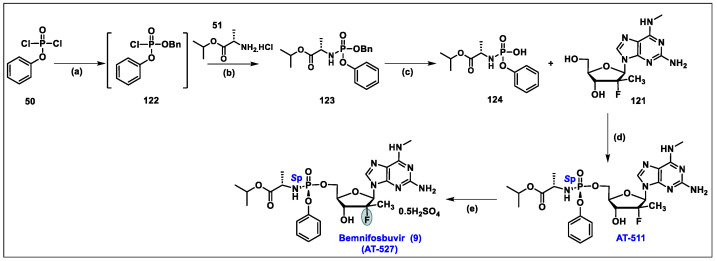
Synthesis of bemnifosbuvir (**AT-527**, **9**) and **AT-511**. (**a**) BnOH, NEt_3_, isopropyl acetate; (**b**) L-alanine isopropyl ester. HCl, NEt_3_, isopropyl acetate; (**c**) IPA, dihydroquinine, 5% Pd/C, H_2_, 1 atmospheric pressure; (**d**) (i) HATU, DIPEA, DCM; (**e**) H_2_SO_4_.

The synthesis of the phosphoramidate prodrug, bemnifosbuvir (AT-527, **9**), is shown in Scheme 19. Phenyl dichlorophosphate **50** was reacted with benzyl alcohol in the presence of triethylamine (NEt_3_) in isopropyl acetate to give intermediate **122,** which on further reaction with L-alanine isopropyl ester hydrochloride (**51**) constructed compound **123**. The debenzylation of **123** under hydrogenation conditions using quinine and Pd/C afforded intermediate **124** as a dihydroquinine salt. The coupling of intermediate **124** with nucleoside **121** was carried out with HATU/DIPEA in DCM to yield AT-511, which after isolation was triturated with isopropyl acetate to produce the diastereomerically pure *S*p isomer of phosphoramidate prodrug AT-511. The sulfuric acid salt of AT-511 was prepared by treating it with con. H_2_SO_4_ to give bemnifosbuvir (AT-527, **9**) as a sulfate salt [143]. 

Bemnifosbuvir has expressed potent anti-HCV activity in the preliminary in vitro screening. The EC_50_ of AT-511 was determined against the various laboratory strains of HCV and clinical isolates with genotype 1–5 and was found in a range of 5–28 nM. Additionally, AT-511 exhibited 10-fold more antiviral activity than sofosbuvir against various laboratory strains and clinical isolates of HCV genotypes 1–5. This molecule also retained its activity against S282T resistance-associated variants and was 58-fold more potent than sofosbuvir without causing human DNA toxicity [139]. The triphosphate of AT-527 is active metabolite, which readily generates from its phosphoramidate prodrug, and has a 10 h half-life in hepatocytes. When orally administered AT-527 in rats and monkeys, it preferentially produces a high concentration of triphosphates in the liver. These beneficial clinical properties of bemnifosbuvir support its ongoing clinical development and suggest that it may increase the sustained viral response (SVR) rate in combination with other classes of anti-HCV drugs. In combination, AT-527 may potentially shorten the treatment duration for patients infected with HCV. Amid the COVID-19 pandemic, the free base AT-511 was tested against several coronaviruses, including SARS-CoV-2. In human airway epithelium (HAE) cells, AT-511 demonstrated EC_90_ of 0.47 µM, which was very similar to its EC_90_ against human coronavirus (HCoV)-229E, HCoV-OC43, and SARS-CoV-2 in Huh-7 cells. Across various coronaviruses, AT-511 has shown multiple different micromolar EC_50_ and EC_90_ values. This includes HCoV-229E (EC_50_ = 1.8 μM), HCoV-OC43 (EC_90_ = 0.5 μM), MERS-CoV (EC_50_ = 26 μM), SARS-CoV-2 (EC_90_ = 0.47 μM), and SARS-CoV (EC_90_ = 0.34 μM, Table 8) [140]. Additionally, an overall high selectivity of AT-511 was observed, with CC_50_ values ranging from 86 to >100 μM depending on the cell line [140].

Pharmacokinetic studies indicated that administering AT-527 orally at 550 mg twice in nonhuman primates produces an efficient concentration of its active triphosphate form in the lungs and effectively inhibits the replication of SARS-CoV-2. This finding indicates that AT-527 holds promise as a clinical candidate for treating COVID-19 infections. Currently, AT-527 is in phase 2 investigation for the possible treatment of SARS-CoV-2 infection [144]. 

### 5.10. AT-752 (10)

**Figure 8:**
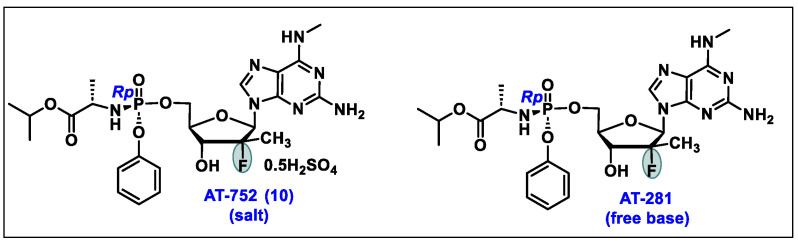
Structures of **AT-752** hemisulfate salt and its free base **AT-281**.

AT-752 is a hemisulfate salt of **AT-281**’s free base (Figure 8) and has potent in vitro activity against dengue virus 2 (DENV 2) and DENV 3 serotypes, including all other flaviviruses. AT-752 is an orally available double prodrug of a guanosine nucleotide, currently under phase 2 pre-clinical development [145]. **AT-281** is a congener of **AT-511** with a *R*p stereocenter at phosphorous, while **AT-511** has a *S*p stereocenter. In vitro, in Huh 7.5 cells, AT-281 demonstrates EC_50_ values of 0.48 and 0.77 μM against DENV serotypes 2 and 3, respectively. AT-281 also exhibited potent antiviral activity against other flaviviruses, with EC_50_ values ranging from 0.19 to 1.41 μM without expressing cytotoxicity at concentrations up to 170 μM. AT-281 goes through the same metabolic pathway as AT-527, intracellularly in peripheral blood mononuclear cells of mice, rats, and monkeys; it converts to its active triphosphate and inhibits DENV2 RNA polymerase. RNA polymerase is essential for viral replication; the inhibition of this enzyme by active AT-281 triphosphate and its incorporation in the prolongating RNA chain results in the termination of viral RNA synthesis. Thus, it is considered a direct-acting antiviral (DAA) [146]. So far, the revealed in vitro and in vivo activity of AT-752 indicates that it is a promising clinical candidate for the treatment of dengue virus infection and is currently under evaluation in clinical studies [147]. Furthermore, recently, Kai et al. reported that AT-752 has potential in vitro activity against yellow fever virus (YFV) [148]. 

### 5.11. AL-335 (11)


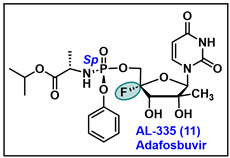


AL-335 is a next-generation investigational HCV therapeutic nucleotide analog developed by Alios Biopharma. Currently, it is under phase 2 clinical trials as a mono-therapeutic agent (clinical trials.gov number: NCT02339207) [149] or in combination with simeprevir and odalasvir (clinical trials.gov number: NCT02569710) [150]. AL-335 is a phosphoramidate prodrug and a modified ribose analog of uridine with 4′-fluoro-2′-*α*-hydroxy-2′-*β*-methyl sugar moiety. AL-335 inhibits HCV nonstructural protein NS5B and in vitro expressed EC_50_ values ranging from 0.04 to 0.06 µM [151]. It acts as a DAA by inhibiting viral RdRp via chain termination without causing any cytotoxicity to the host and demonstrates in vitro and in vivo a better safety and efficacy profile in comparison to currently used drugs [152]. In the subgenomic HCV replicon assay, the *Sp* isomer of AL-335 (EC_50_ = 0.07 µM) was found 13-fold more active than the *R*p isomer (EC_50_ = 0.94 µM).

**Scheme 20:**
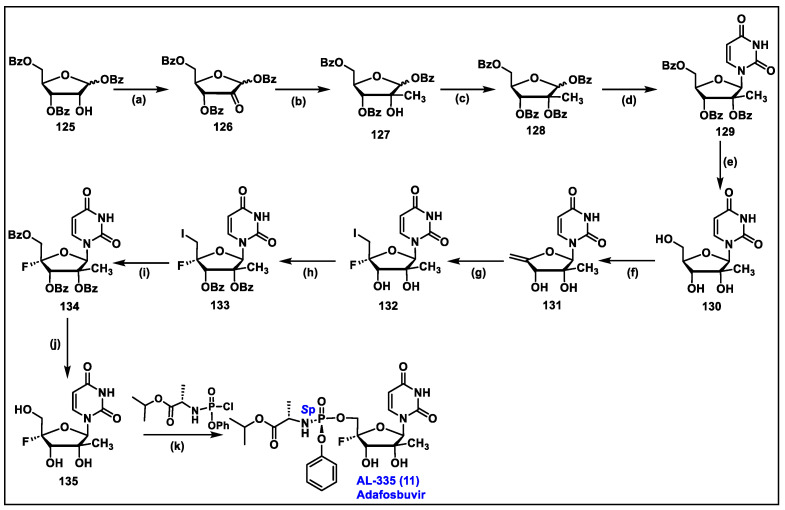
Synthesis of **AL-335** from intermediate **126**. (**a**) Dess–Martin periodinane, DCM; (**b**) MeMgBr, −70 to −40 °C, THF; (**c**) BzCl, DMAP, NEt_3_, DCM; (**d**) uracil, *N*,*O*-bis(trimethylsilyl)acetamide, SnCl_4_; (**e**) NH_3_, MeOH; (**f**) (i) I_2_, imidazole, pyridine, THF; (ii) DBU, THF; (**g**) NIS, 3HF.NEt_3_; (**h**) BzCl, DMAP, DCM; (**i**) NaOBz, 15-crown-5, DMSO; (**j**) MeNH_2_, EtOH; (**k**) (i) NMI, THF; (ii) prep HPLC separation.

Wang et al. reported a range of uridine analogs featuring diverse sugar-modified nucleoside substitutions at the 4′-F-2′-C positions. Among these, AL-335 emerged as a prominent nucleotide candidate effective against HCV. The synthesis of AL-335 was initiated with commercially available intermediate **125** as shown in Scheme 20 [151,153]. The oxidation of 2-hydroxy of **125** with Dess–Martin periodinane gives 2-keto ribofuranose **126**. Intermediate **126** was treated with MeMgBr to yield **127**, which on treatment with benzoyl chloride, in the presence of catalytic amount of DMAP with NEt_3_ in DCM to afford tetra benzoate sugar **128**.

Compound **128** was coupled with silylated uracil base under Vorbrüggen glycosylation conditions to give the desired intermediate **129**. The deprotection of benzoyl group of **129** was carried out under a basic condition to afford 2′-*C*-substituted uridine **130**. Next, to install fluoro at the 4′-position of uridine **130**, it was treated with I_2_ under basic conditions to afford 4′,5′-alkene intermediate **131**. Compound **131** was further treated with NIS and 3HF.NEt_3_ to produce 4′-F,5′-iodo intermediate **132**. The benzoylation of **132** was followed by the substitution of iodo with sodium benzoate to obtain compound **134.** Furthermore, the debenzylation of **134** with MeNH_2_ produces the key nucleoside **135** [154]. Compound **135** was coupled with isopropyl (chloro(phenoxy)phosphoryl)-L-alaninate in the presence of NMI furnishes racemic phosphoramidate prodrug, which on chiral separation vis HPLC affords the desired *Sp* isomer of AL-335 [151]. 

AL-335 has expressed potent activity against the wild-type (WT-GT1b) HCV EC_50_ value of 0.04 µM and was further tested against various HCV genotypes. As shown in Table 9, AL-335 retained a potent antiviral activity against all HCV genotypes GT1a, GT1b, GT2b, GT3b, and GT4a, with EC_50_ values ranging from 0.04 to 0.06 μM. In addition, it did not show any significant cytotoxicity in multiple cell lines as depicted in Table 10.

### 5.12. FMCAP (12)

**Figure 9:**
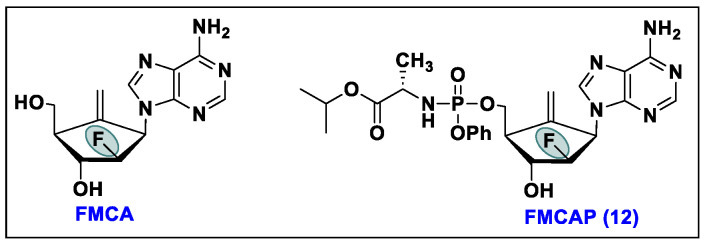
Structures of FMCA and its phosphoramidate prodrug FMCAP.

FMCA is chemically known as 2′-fluoro-6′-methylene carbocyclic adenosine. To improve the antiviral potency of FMCA, its phosphoramidate prodrug FMCAP (Figure 9) was invented. Both these molecules have demonstrated potent activity against drug-resistant HBV and are currently in preclinical evaluation for the treatment of chronic hepatitis B (CHB) [155]. These molecules belong to a special class of nucleoside moiety called carbocyclic nucleosides. In a carbocyclic nucleoside, the oxygen of the five-membered sugar ring is replaced with a carbon atom, which provides enzymatic and metabolic stability to the glycosidic bond. FMCA is a mimetic analog of the anti-HBV drug entecavir, in which the 2′-position of a carbocyclic moiety contains a β-fluorine atom with an adenine base in place of guanine. The design, synthesis, and antiviral evaluation of FMCA also proves the concept that the insertion of fluorine at the 2′-position of the nucleoside strengthens the glycosidic bond toward metabolic and chemical degradation. It may maintain additional hydrogen bonding/or interaction in the binding pocket of viral polymerase. The incorporation of the 2′-fluorine in FMCAP was advantageous to antiviral activity, and it has demonstrated promising anti-HBV activity against lamivudine/entecavir triple mutants (L180M + S202G + M204V) [156]. To cross the rate-limiting first step monophosphorylation, the phosphoramidate prodrug (FMCAP) of FMCA was synthesized, which expressed 10 times more potency than the parental molecule [156].

The initial synthesis of FMCA was started with carbocyclic ketone **136**, which was synthesized in nine steps from D-ribose [157,158]. The sixth position methylene was added to ketone **136** by treating it with lithium diisopropylamide (LDA) and Eschenmoser’s salt and methyl iodide, which on selective reduction under Luche reduction conditions furnished allylic alcohol **137** [159]. The allylic hydroxyl group of **137** was protected with a benzyl group to give **138** that, on subsequent deprotection of acetonide and the *tert*-butyl group, produces triol **139**. The selective protection of the 3,5-hydroxy groups of **139** with 1,3-dichloro-1,1,2,2-tetraisopropyl disilazine (TIPDSCl_2_) afforded compound **140**.

**Scheme 21:**
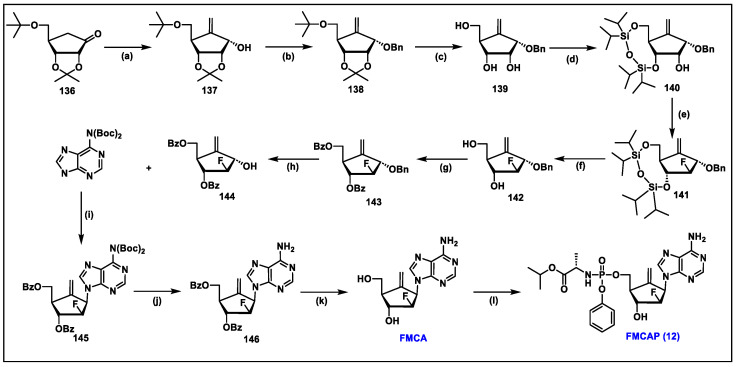
Synthesis of FMCA and its prodrug FMCAP, **12** via carbocyclic ketone **136**. (**a**) (i) LDA, Eschenmoser’s salt; (ii) MeI; (iii) NaHCO_3_; (iv) NaBH_4_/CeCl·7H_2_O; (**b**) NaH, BnBr, DMF; (**c**) TFA/H_2_O; (**d**) TIDPSCl_2_/imidazole, DMF; (**e**) DAST, DCM; (**f**) TBAF, AcOH, THF; (**g**) BzCl, pyridine; (**h**) BCl_3_, DCM; (**i**) 6-*N*,*N*-diboc adenine, DIAD, PPh_3_, THF; (**j**) TFA/DCM; (**k**) DIBAL-H, DCM; (**l**) L-alanine isopropyl ester chlorophosphoramidate, NMI, THF.

Intermediate **140** was treated with diethylaminosulfur trifluoride (DAST) to convert the 2-*α*-hydroxyl group of **140** to the 2-*β*-fluoro intermediate **141** via an S_N_2 mechanism. The silyl deprotection of **141** with TBAF/AcOH in DCM produces **142**, which on reprotection with a benzoyl group constructed compound **143**. The debenzylation of **143** afforded key intermediate **144**, which was further coupled with *N,N*-diboc-protected adenine under Mitsunobu conditions to render coupled intermediate **145**. After the benzoyl and boc deprotection of **145**, the final nucleoside FMCA was furnished (Scheme 21) [155,160]. Finally, the phosphoramidate prodrug, FMCAP, was synthesized through the coupling of FMCA with the L-alanine isopropyl ester chlorophosphoramidate reagent in THF in basic conditions using *N*-methyl imidazole (NMI) to afford the phosphoramidate prodrug FMCAP, 12 in good yield [155,156].

**Scheme 22:**
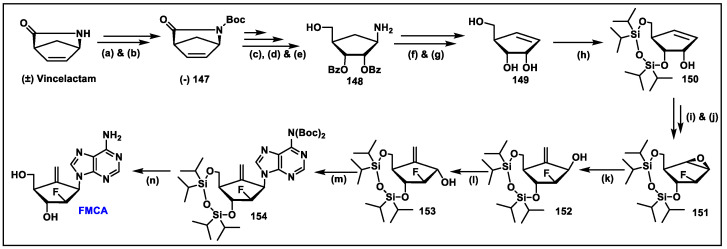
Synthesis of FMCA via Vince lactam. (**a**) (Boc)_2_O, DMAP, THF, rt; (**b**) savinase, THF, buffer solution, rt; (**c**) OsO_4_, NMO, acetone, rt; (**d**) (i) BzCl, DMAP, DCM; (ii) NaBH_4_, MeOH, rt; (**e**) HCl/ether, MeOH, rt; (**f**) NaNO_2_, acetic acid, water, acetonitrile; (**g**) NaOMe, MeOH, rt; (**h**) TIPDSCl_2_, imidazole, DMF; (**i**) *m*-CPBA, DCM, rt; (**j**) DAST, DCM; (**k**) *n*-BuLi, trimethylsulfonium iodide, THF; (**l**) (i) Dess–Martin periodinane, DCM, rt; (ii) NaBH_4_, CeCl_3_.7H_2_O, MeOH; (**m**) *N’N*-diBoc-adenine, DIAD, TPP, THF, rt; (**n**) TFA, TBAF/THF, rt.

The preliminary antiviral results of FMCA and FMCAP have demonstrated effective anti-HBV activity against both wild-type and drug-resistant HBV mutants. To overcome the lengthy synthetic steps in the original synthetic method described in Scheme 21, a new synthetic methodology, in 16 steps, was developed for the synthesis of FMCA from Vince lactam, as shown in Scheme 22 [161]. 

The *N*-Boc protection of a commercially available (±) γ-lactam yielded Boc-protected (±) γ-lactam, which, on the enzymatic resolution, produced a chiral pure (-) γ-lactam **147**. The resolution of Boc-protected (±) γ-lactam was performed by savinase in 50% THF-Buffer solution (pH 8.0) to afford optically pure (-) γ-lactam **147** with an enantiomeric excess (*ee*) of more than 99%. The hydroxylation of optically pure (-) γ-lactam **147** was carried out with OsO_4_/NMO to furnish the -diol. Subsequently in terms of benzyl protection was performed followed by the opening of the lactam with sodium borohydride (NaBH_4_) and the deprotection of *N*-Boc with 2 M solution of HCl/ether yielded intermediate amine **148** as an hydrochloric salt. Diazotization followed by the elimination of the amine **148** gave the alkene intermediate. Further, the deprotection of the benzoyl group of the alkene intermediate with sodium methoxide yielded triol **149**. The 3- and 5-hydroxyl groups of triol **149** were selectively protected with TIPDSCl_2_ in DMF to give compound **150**. The epoxidation of the alkene was followed by the fluorination of **150** with DAST furnished *β*-fluoro compound **151**. The selective opening of the epoxide **151** with dimethylsulfonium methylide yielded the *β*-allylic alcohol **152**. The inversion of the hydroxyl group of **152** via oxidation followed by the Luche reduction [162] generated **153**. The key intermediate 153 was coupled with Boc-protected adenine under Mitsunobu conditions to produce the coupled nucleoside 154. The deprotection of silyl and Boc-groups of 154 generated the final target compound FMCA in moderate yield.

The synthesis of FMCA described in Scheme 22 from Vince lactam was reasonable for a medium scale synthesis (2–10 gm) [161]. However, due to the overall low yield, this process was not viable for the large-scale synthesis of FMCA. A significant drawback of Scheme 22 was the low yield of diazotization elimination, as well as the inversion of a hydroxyl group of 152. Furthermore, certain costly reagents and materials were required in the process, which makes the process unattractive for large-scale synthesis of FMCA. To overcome the drawback associated with Scheme 22, Singh et al. developed another synthetic route with fewer steps and milder conditions [163]. The revised synthesis was again started with the carbocyclic ketone 136. The sixth position methylene group was inserted by treating ketone 136 with a mixture of paraformaldehyde and diisopropylamine TFA salt, followed by a selective Luche reduction to give allylic alcohol 137. The selective isopropylidene opening of **137** was carried out with trimethylaluminum in hexane to produce 2,3-dihydroxy compound **155**. The selective protection of the first-positioned hydroxy of **155** was performed with a TBDPS group to afford intermediate **156**.

**Scheme 23:**
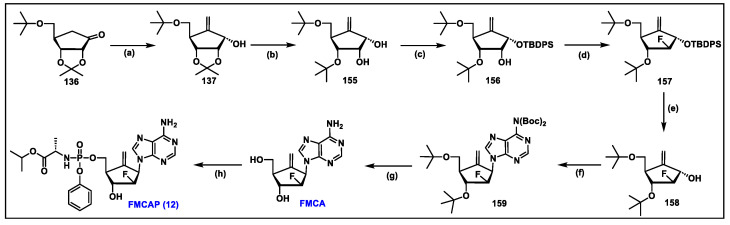
Revised scalable synthesis of FMCAP from ketone **156**. (**a**) (i) (HCHO)*n*, *i*-Pr_2_NH.TFA, diisopropylamine, THF; (ii) NaBH_4_, CeCl_3_⋅7H_2_O, MeOH; (**b**) Al(Me)_3_ (2.0 M in hexane), DCM; (**c**) TBDPSCl, imidazole, DCM; (**d**) DAST, DCM; (**e**) TBAF, THF; (**f**) 6-*N*,*N*-diboc-adenine, DIAD, PPh_3_, THF; (**g**) TFA, DCM; (**h**) L-alanine isopropyl ester chlorophosphoramidate, NMI, THF.

The fluorination of the 2-hydroxy of **156** was accomplished with DAST to give the fluorinated compound **157**. The deprotection of TBDPS afforded hydroxy key intermediate **158**, which, on coupling with 6-*N*,*N*-diboc adenine under Mitsunobu coupling, furnished coupled nucleoside **159**. The final deprotection of the protecting groups of **159** by 2 M solution of TFA in DCM afforded FMCA (Scheme 23) [163]. The condensation of FMCA with L-alanine isopropyl ester chlorophosphoramidate in the presence of NMI gave phosphoramidate prodrug FMCAP, **12**. The synthetic route of FMCAP explained in Scheme 23 utilizes fewer steps with more economical reagents that enhance the relevancy of synthetic route for large-scale synthesis.

FMCA demonstrated significant antiviral activity against the wild-type, as well as adefovir- and lamivudine-resistant mutants HBV (Table 11). 

FMCA showed antiviral activity against wild-type (WT) HBV with an EC_50_ value of 1.5 µM. In WT, its antiviral potency was analogous to adefovir, while it was 7-fold less potent than lamivudine. The concentration of FMCA required to inhibit 90% (EC_90_) of wild-type HBV is 4.5 µM, which is 1.5-fold more potent than adefovir (EC_50_ = 7.1 µM). However, it is important to note that FMCA was more active against both lamivudine- and adefovir-associated HBV mutants. FMCA demonstrated that it is 4.5-fold more potent than adefovir for the EC_50_ value (1.7 µM) and 7.8-fold more potent for EC_90_ value (4.6 µM), respectively, against adefovir mutant rtN236T. For rtM204V, FMCA showed a similar EC_50_ value with respect to adefovir, while, in rtM204I, FMCA showed an EC_50_ of 1.0 µM that is approximately 2-fold more potent than adefovir. For the rtM204I mutant, FMCA also exhibited higher potency with an EC_90_ 5.0 µM.

For the mutant rtL180M, the antiviral activity of FMCA exhibited similarly to that of lamivudine in EC_50_ value (2.1 vs. 1.5), while for the EC_90_ (5.1 vs. 22.0) value, it showed a 4.3-fold increased antiviral activity. However, in the case of the double mutant rtL180M/rtM204V, FMCA exhibited a similar EC_50_ value (2.2 µM) to adefovir, but it showed a superior EC_90_ value (5.5 µM) than adefovir (8.5 µM).

Furthermore, FMCA and its prodrug FMCAP were screened against the wild-type, as well as the lamivudine-entecavir resistant clone (L180M + M204V + S202G). The results are listed in Table 12 [156]. 

FMCA and FMCAP exhibited significant anti-HBV activity with an EC_50_ of 0.548 ± 0.056 and 0.062 ± 0.011 µM, respectively, against the wild-type virus (Table 12). In the wild type, FMCAP exhibited an eight-fold enhanced activity compared to FMCA.

The further antiviral evaluation of FMCA and FMCAP in vitro activity against the triple resistant lamivudine-entecavir clone (L180M + M204V + S202G) demonstrated an EC_50_ of 0.67 and 0.054 µM, respectively, which confirmed the antiviral potency of these preclinical candidates against the drug-resistant mutants. In the drug-resistant clone (L180M + M204V + S202G), FMCAP was found 12 times more potent than FMCA, while entecavir lost its potency by 150-fold in comparison to its wild type and lamivudine exhibited complete incompetence against the resistant clone [164]. The preliminary in vivo studies in chimeric mice having the lamivudine/entecavir triple mutants FMCA and FMCAP reduced HBV viral load, whereas entecavir was found ineffective. Also, in female NOD/SCID mouse models, these molecules showed a higher rate of reduction in liver HBV DNA levels in comparison to entecavir [165]. 

### 5.13. NITD-17 (13)

In 2010, Ganapati et al. reported cyclic prodrugs of 2′-deoxy-2′-fluoro-2′-*β*-*C*-methyl guanosine PSI-352938 [137] and Chang et al. described linear phosphoramidate PSI-353661 [166]; these analogs have demonstrated strong potent HCV inhibitors [137]. 

**Figure 10:**
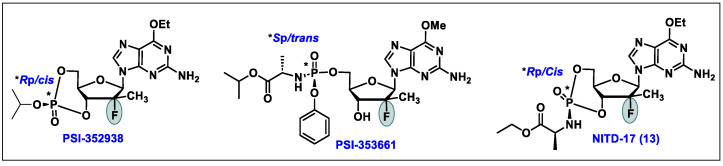
Structures of PSI-352938, PSI-353661 and NITD-**17** (**13**).

Encouraged by these antiviral activities of common guanosine analogs, Karuna et al. described a cyclic phosphoramidate prodrug of 2′-deoxy-2-*β*-fluoro-2′-*C*-methylguanosine (NITD-**17**, Figure 10) that in vitro and in vivo demonstrated excellent activity against dengue virus (DENV). Intracellular enzymes unmask the prodrug NITD-**17 (13)** and metabolize it into an active triphosphate form to inhibit viral replication. To identify a potent analog against the dengue virus, more than 150 cyclic phosphoramidate prodrugs were prepared with variations in the amino acid ester and *C*-6 position of the guanosine base. Among these, it was concluded that cyclic phosphoramidate prodrugs have several advantages over linear phosphoramidates. Cyclic phosphoramidate prodrug strategies also mask polar 3′-OH, reduce the degree of rotational freedom of molecule, and enhance cell permeability and cellular uptake. Cyclic prodrugs further offer metabolic stability in the liver and alleviate the circulation of drug molecules in other parts of the body. Keen, using these explained findings, discovered NITD-**17** (**13**) to cure dengue infection [167]. 

**Scheme 24:**
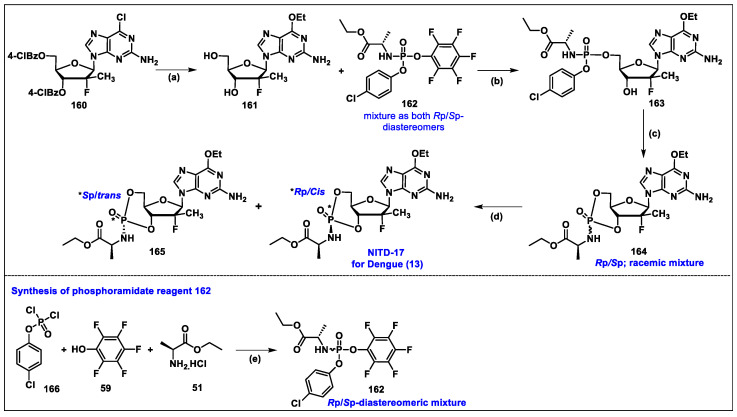
Synthesis of NITD-**17** from compound **160**. (**a**) NaOEt, EtOH; (**b**) reagent **162**, *t*-BuMgCl in THF; (**c**) *^t^*BuOK, DMSO; (**d**) separation of pure isomer by reverse phase prep HPLC; (**e**) NEt_3_, DCM.

The synthesis of NITD-**17** has been described in Scheme 24 from compound **160**. Compound **160** was treated with 21% wt. solution of sodium ethoxide to obtain guanosine intermediate **161**. Nucleoside **161** was further coupled with pentafluorophenyl phosphoramidate reagent **162** under basic conditions using *t*-BuMgCl to afford linear phosphoramidate prodrug **163**, which was further treated with potassium *tert.* butoxide to produce a racemic mixture of cyclic phosphoramidate prodrug **164**.

The chiral pure cyclic phosphoramidate prodrug NITD-**17** was obtained via a preparative HPLC separation that afforded the desired chiral pure *R*p isomer (*cis* configuration at phosphorous center) of NITD-**17**. Furthermore, the *R*p stereochemistry of NITD-**17** was confirmed via single-crystal X-ray data analysis. Additionally, it was found that in the ^31^P-NMR analysis, the phosphorous peak of the *R*p isomer of NITD-**17** appears in the higher field region than its congener *S*p (*trans* configuration at phosphorous center) isomer **165**.

NITD-**17** expressed pan-serotype and good antiviral activities in multiple cell lines. The antiviral activities of NITD-**17** were examined in primary human PBMCs and other cell lines against all four DENV serotypes (Table 13). NITD-**17** demonstrated potent antiviral activity against all DENV serotypes. In the PBMC cell line, NITD-**17** showed EC_50_ values of 0.18, 0.23, 0.36, and 0.37 µM against DENV serotype 1–4, respectively. An EC_50_ of 0.46 µM was obtained in the THP-1 cell line against DENV-2. In the DENV-2 replicon assay in the Huh 7 cell line, it demonstrated 1.73 µM activity with cellular toxicity up to >100 µM (CC_50_).

NITD-**17** expressed significant activity in various cell lines, which prompted further evaluation of its in vivo efficacy against DENV infection had exhibited a broad tissue tropism. During the preclinical study, it was proven that NITD-17 converted to the active triphosphate form in PBMCs across multiple species, and in in vivo studies in an AG129 mouse model, it effectively reduces 1.6- and 2.2-log viremia at 100 and 300 mg/kg twice a day (BID). Later, during the toxicological assessment, this molecule expressed pulmonary inflammation and hemorrhage and was discontinued for further preclinical development [167]. 

### 5.14. AL-611 (14)


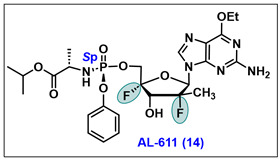


AL-611 was developed by Janssen Biopharma (formerly Alios) to treat chronic hepatitis C (CHC). In oral treatment, AL-611 in dogs demonstrated a high concentration of AL-611-triphosphate (TP). AL-611 expressed an excellent anti-HCV activity and was selected as a clinical candidate. Later, it was discontinued due to the similar effectiveness of already approved drugs. AL-611 is a guanosine phosphoramidate prodrug and contains a modified sugar moiety identical to sofosbuvir with an additional 4′-fluoro-substitution on the sugar ring. AL-611 design demonstrates a fixed base and altered sugar approach of nucleosides. AL-611 exhibited potent antiviral activity in its triphosphate form by inhibiting the HCV NS5B polymerase. Due to its potent in vitro activity as indicated by its EC_50_ value of 5 nM against viral HCV polymerase, the researchers at Janssen BioPharma aimed to develop AL-611 as a safe, effective, and shortened treatment as a HCV cure [168]. Unfortunately, it was halted in phase 1 clinical trials because the overall efficacy and effectiveness of AL-611 were not superior when compared to other prescribed drugs.

**Scheme 25:**
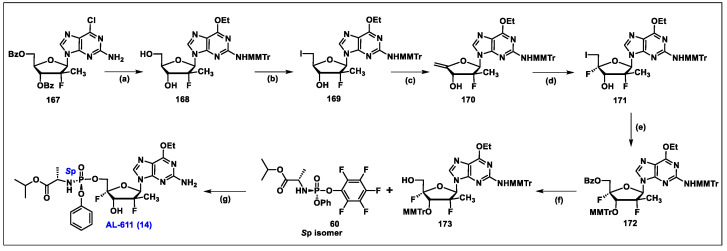
Synthesis of AL-611 from nucleoside **167.** (**a**) (i) MMTrCl, AgNO_3_, collidine, DCM; (ii) NaOEt, EtOH; (**b**) I_2_, PPh_3_, imidazole, THF; (**c**) DBU, THF; (**d**) NEt_3_.3HF, NIS; (**e**) (i) MMTrCl, AgNO_3_, collidine, DCM, rt; (ii) NaOBz, DMF, 15-crown-5; (**f**) n-butylamine, rt; (**g**) (i) *t*-BuMgCl, THF; (ii) HCl/CH_3_CN.

Nucleoside **167** was synthesized from the reported protocol (Scheme 25) [137]. First, 2-amino of **167** was protected with monomethoxytrityl; after that, installation of 6-*O*-ethyl was carried out by treating it with sodium ethoxide (NaOEt) in EtOH to give debenzylated compound 168. Furthermore, the 5′-hydroxy of **168** was converted to 5′-iodo compound **169**. Compound **168** was treated with iodine in the presence of TPP/imidazole in THF to produce **169**, which, on treatment with DBU in THF, furnished essential alkene nucleoside **170**.

The insertion of 4′-fluoro was accomplished by the reaction of N-iodosuccinimide (NIS) and triethylamine trihydrofluoride with the alkene of 170 to produce 5′-iodo-4′-fluoro nucleoside analog 171 as a single isomer. The further protection of 3′-OH of **171** with monomethyoxytrityl followed by the replacement of iodo with *O*-benzoyl afforded nucleoside 172. The debenzylation of **172** with *n*-butylamine produced the key nucleoside **173**. The coupling of **173** with phosphoramidate reagent **60** in the presence of *t-*BuMgCl in THF followed by the deprotection of the 3′-trityl group afforded the targeted phosphoramidate prodrug AL-611, **14** [168].

In an HCV replicon assay against the NS5B polymerase, AL-611 showed nanomolar potency, an EC_50_ of 5 nM, and a subsequent cytotoxicity CC_50_ value of >100 μM, having a respective SI greater than 20,000 (Table 14) [168]. 

## 6. Conclusions

Nucleos(t)ides (NAs) are widely known for their antiviral and anticancer potency; among them, fluoro-containing nucleos(t)ides exert more prominent clinical candidates than their non-fluoro parent counterparts. Several fluoro-containing nuclosi(t)des have emerged as FDA-approved drugs for the treatment of viral infections. The insertion of fluorine/CF_3_ at the sugar or bases moiety of a nucleoside and the installation of 5-F/CF_3_, especially at the pyrimidine ring of nucleosides, have resulted in the invention of numerous antiviral drug candidates. The therapeutic molecules of this class selectively and specifically target the viral DNA/RNA polymerase and inhibit viral growth. This review article elucidates the synthesis and antiviral activity of FDA-approved fluro-nucleos(t)ide drugs and covers various fluoro nucleosides, which are at the various stages of clinical development as antiviral agents.

## Figures and Tables

**Table 1:** Emtricitabine (FTC) antiviral activity against laboratory-adopted HIV strains in the human cell lines.

HIV Strains	Cells Line	EC_50_ (µM)					Ref.
		FTC	3TC	AZT	ddl	ddc	
HIV-1IIIB	CEM	0.100	0.30	0.030	8.5	0.03	[55]
	MT-4	0.500	3.20	0.060	16.0	0.05	
HIV-1LAV	CEM	0.009	0.07	0.005	NA	NA	
	HT4-6C	0.020	NA	NA	NA	NA	
HIV-2ZY	CEM	0.100	0.30	0.030	NA	NA	
	MT4	1.500	9.80	0.040	NA	0.04	

EC_50_: 50% effective concentration; FTC, emtricitabine; 3TC, lamivudine; AZT, zidovudine; ddl, didanosine; ddc, zalacitabine; CEM, and MT-4, human T-lymphoblastoid cell lines; HT4-6C, Cd4-expressing HeLa Cell line; NA, not available.

**Table 2:** In vitro antiviral activity of sofosbuvir against different viruses.

Viruses	Cells	EC_50_ or IC_50_	CC_50_	Ref.
Zika	Huh7	IC_50_ = 4.1 µM	>100 µM	[89]
HCV	HCV Replicon Assays—full-length replicons from genotype 1a, 1b, 2a, 3a and 4a, and chimeric 1b replicons encoding NS5B from genotype 2b, 5a or 6a	EC_50_ = 0.014–0.11 µM	>100 µM	[87]
West Nile	Huh7	IC_50_ = 1.2 ± 0.3 µM	>400 µM	[90]
Dengue	Huh7	EC_50_ = 4.9 ± 1.3 µM	>100 µM	[91]

EC_50_: 50% effective concentration; CC_50_: 50% effective cytotoxic concentration.

**Table 3:** In vitro antiviral activity of clevudine against HBV and EBV.

Compound	Anti-HBV Activity in 2.2.15 Cells (EC_50_, µM)	Anti-HBV Activity in HepAD38 Cells (EC_50_ µM)	Anti-EBV Activity in H1 Cells (EC_90_, µM)	Anti-EBV Activity in P3HR1 Cells (EC_90_, µM)	HBV Growth Inhibition (ID50, µM) within the Following Cells:				Ref.
					MT2	CEM	H1	2.2.15	
Clevudine	0.1	0.11 ± 0.04	5 ± 0.8	5.0 µM	100	>100	913 ± 70	>200	[92]

EC_50_ = 50% effective concentration. EC_90_ = 90% inhibitory concentration determined by bioassay. ID_50_ = 50% inhibitory dose.

**Table 4:** In vitro antiviral activity of elvucitabine.

Compound	Virus	Cells	EC_50_	CC_50_	Ref.
Elvucitabine	HBV	CEM	0.008 μM	7 μM	[108]
	HIV	CEM	0.15 μM	7 μM	[108]
	HIV	PBM	0.034 μM	>100 μM	[109]

EC_50_ = 50% effective concentration. CC_50_ = 50% effective cytotoxic concentration.

**Table 5:** Antiviral activity of fiacitabine (FIAC) against various viruses.

Viruses	In Vitro Cells of Screening	Antiviral Activity in (µM)	Ref.
HSV-1 and 2	Vero cells	ED_50_ = 0.01 µM	[111]
Equine rhinopneumonitis (EHV-1)	(rabbit kidney) RK13 monolayer	IC_50_ = 0.09–0.18 µM	[112]
Aujeszky’s disease virus (SHV-1)	(rabbit kidney) RK13 monolayer	IC_50_ = 0.25–7.0 µM	[112]
Bovine rhinotracheitis virus (BHV-1)	(rabbit kidney) RK_13_ monolayer	IC_50_ = 0.1–3.0 µM	[112]
EBV	P3HR-1 cells	EC_50_ = 0.4 ± 0.3 µM	[113]
KSHV	BCBL-1 cells	EC50 = 1.1 ± −0.8 µM	[113]
Human cytomegalovirus (HCMV)	human foreskin fibroblasts	ED50 = 0.6 ± 0.13 µM	[114]

ED_50_: indicates the effective dose to suppress viral replication by 50% (HSV-1, strain 2391; HSV-2, strain G). IC_50_: indicates the inhibitory concentration necessary for 50% inhibition of growth of viruses. EC_50_: indicated 50% effective concentration: the drug concentration required to reduce 50% of cytopathic effect (CPE) or viral DNA copies.

**Table 6:** In vitro anti-HIV activity of islatravir in MT-4 cell line against wild-type and drug resistant HIV.

Compound	Virus	Cells	EC_50_	CC_50_	Ref.
Islatravir	WT HIV	MT-4	0.068 nM	7500 nM	[123]
	M184V HIV	MT-4	3.1 nM	7500 nM	[123]
	MDR HIV	MT-4	0.15 nM	7500 nM	[123]

EC_50_: inhibitory concentration; CC_50_: cytotoxicity concentration.

**Table 7:** In vitro activity of mericitabine in various HCV assays.

Compound	Virus	Cells	IC_50_	Ref.
PSI-6130-TP (RO4995855-TP)	HCV	Huh7 cells containing a sub genomic genotype 1b Con1 strain HCV replicon	0.13 ± 0.001 µM	[82]
	HCV	native HCV replicase complex isolated from replicon cell	0.34 µM	[135]
	HCV	recombinant RdRp in a cell-free enzyme assay (HCV)	0.13 µM	[135]

PSI-6130-TP: PSI-6130-triphosphate. IC_50_: 50% inhibitory concentration.

**Table 8:** In vitro activity of **AT-511** against various human coronaviruses.

Compound	Virus	Cell Line	CPE Assay EC_50_ or EC_90_	CC_50_	Ref.
AT-511	HCoV-229E	BHK-21	EC_50_ = 1.8 μM	>100 μM	[140]
	HCoV-OC43	Huh7	EC_90_ = 0.5 μM	>86 μM	[140]
	MERS-CoV	Huh7	EC_50_ = 26 μM	>86 μM	[140]
	SARS-CoV-2	HAE	EC_90_ = 0.47 μM	>86 μM	[140]
	SARS-CoV	Huh7	EC_90_ = 0.34 μM	>86 μM	[140]

The antiviral activity of **AT-511** was measured in cells infected with different coronaviruses using the cytopathic effect (CEP neutral red dye) assay. EC_50_ = 50% inhibition of the virus induced cytopathic effect (CPE). EC_90_ = 90% inhibition of the virus induced cytopathic effect (CPE). CC_50_ = drug to cause death to 50% of viable cells without virus.

**Table 9:** HCV pan-genotypic replication activity of AL-335.

Compound	Virus Strains	EC_50_ (µM)	Ref.
AL-335	WT-GT1b	0.04 ± 0.02	[151]
	GT1a	0.06 ± 0.01	
	GT2b	0.04 ± 0.01	
	GT3b	0.06 ± 0.01	
	GT4a	0.06 ± 0.01	

EC_50_: 50% effective concentration.

**Table 10:** Results of eight-day cytotoxicity data of AL-335.

Compound	Cell Lines	CC_50_ (µM)	Ref.
AL-335	Hau-7	96.1	[151]
	HepG2	>100	
	A549	>100	
	HeLa	>100	
	U937	>100	
	MT-4	>100	

CC_50_: 50% effective cytotoxic concentration.

**Table 11:** In vitro anti-HBV activity of FMCA against lamivudine and adefovir drug-resistant mutants on the intracellular HBV DNA replication assay.

Strains	FMCA (µM)				Adefovir (µM)			Lamivudine (µM)			Ref.
	EC_50_ ^b^	EC_90_ ^c^	CC_50_ ^d,e^	Fold Resistance (EC_90_)	EC_50_	EC_90_	Fold Resistance (EC_90_)	EC_50_	EC_90_	Fold Resistance (EC_90_)	
Wild Type	1.5	4.5	>100	-	1.3	7.1	-	0.2	0.6	-	[164]
rtM204V	1.8	4.7	>100	1.0	1.6	7.0	1.0	>100	>100	>166	
rtM204I	1.0	5.0	>100	1.1	1.9	8.0	1.1	>100	>100	>166	
rtL180M	2.1	5.1	>100	1.1	5.5	7.7	1.1	1.5	22.0	36.7	
rtLM/rtMV ^a^	2.2	5.5	>100	1.2	2.1	8.5	1.2	>100	>100	>166	
rtN236T	1.7	4.6	>100	1.0	7.8	36.0	5.1	0.2	0.9	1.5	

^a^ rtLM/rtMV = rt180M/rtM204V double mutant. ^b^ Effective concentration required to inhibit 50% of HBV-DNA. ^c^ Concentration required to reduce infectious virus titer by 90%. ^d^ The > sign indicates that the 50% inhibition was not reached at the highest concentration tested. ^e^ The drug concentration required to reduce the viability of cell as determined by MTT assay by 50% of untreated control after 3 days.

**Table 12:** In vitro anti-HBV activity of FMCA and its phosphoramidate (FMCAP) in the correlation of lamivudine and entecavir against wild-type and entecavir drug-resistant mutant (L180M + M204V + S202G) in Huh7 cells.

Compounds	HBV Strains				Ref.
	Wild-Type			L180M + M204V + S202G	
	EC_50_ (µM)	EC_90_ (µM)	CC_50_ (µM)	EC_50_ (µM)	
FMCA	0.548 ± 0.056	6.0 ± 0.400	>300	0.67	[156] [164] [165]
FMCAP	0.062 ± 0.011	0.46 ± 0.060	>300	0.054	
Lamivudine	0.056 ± 0.003	0.142 ± 0.008	>300	>500	
Entecavir	0.008	0.033	28	1.20	

**Table 13:** In vitro activity of NITD-**17** across multiple serotypes and cell lines.

Compound	Cell Type	Assay Type	EC_50_ in µM	Ref.
NITD-**17**	PBMC	DENV-1 (48 h)	0.18 ± 0.06	[167]
		DENV-2 (48 h)	0.23 ± 0.04	
		DENV-3 (48 h)	0.36 ± 0.33	
		DENV-4 (48 h)	0.37 ± 0.14	
	THP-1	DENV-2	0.46 ± 0.20	
	KU812	DENV-2 high content imaging	1.41	
	K562	DENV-2	2.79 ± 0.22	
	293T	DENV-2	3.40	
	Huh7	DENV-2 replicon expressing luciferase	1.73 ± 1.06	

EC_50_: effective concentration of drug for 50% inhibition, PBMC: peripheral blood mononuclear cell; THP-1: human monocyte cells; KU812: basophils that were isolated from the peripheral blood of a 38-year-old Asian male; K562: lymphoblast cells isolated from the bone marrow; 293T: highly transfect derivative of human embryonic kidney 293 cells; Huh7: cell line established from male hepatoma tissue.

**Table 14:** Antiviral potency of **AL-611** against HCV in replicon assay.

Virus	Cells	EC_50_	CC_50_/tox	Ref.
HCV	Replicon Assay	5 nM	>100 µM	[168]

## Data Availability

Not applicable.

## References

[1] De Clercq E., Li G.D. (2016). Approved Antiviral Drugs over the Past 50 Years. Clin. Microbiol. Rev..

[2] Minchin S., Lodge J. (2019). Understanding biochemistry: Structure and function of nucleic acids. Essays Biochem..

[3] Seley-Radtke K.L., Yates M.K. (2018). The evolution of nucleoside analogue antivirals: A review for chemists and non-chemists. Part 1: Early structural modifications to the nucleoside scaffold. Antivir. Res..

[4] Zenchenko A.A., Drenichev M.S., Il’icheva I.A., Mikhailov S.N. (2021). Antiviral and Antimicrobial Nucleoside Derivatives: Structural Features and Mechanisms of Action. Mol. Biol..

[5] Deeks S.G., Overbaugh J., Phillips A., Buchbinder S. (2015). HIV infection. Nat. Rev. Dis. Primers.

[6] Zhou Z., Zheng H., Xiao G., Xie X., Rang J., Peng D. (2024). Effectiveness and safety of azvudine in older adults with mild and moderate COVID-19: A retrospective observational study. BMC Infect. Dis..

[7] Yates M.K., Seley-Radtke K.L. (2019). The evolution of antiviral nucleoside analogues: A review for chemists and non-chemists. Part II: Complex modifications to the nucleoside scaffold. Antivir. Res..

[8] Al Awadh A.A. (2022). Nucleotide and nucleoside-based drugs: Past, present, and future. Saudi. J. Biol. Sci..

[9] Inoue M., Sumii Y., Shibata N. (2020). Contribution of Organofluorine Compounds to Pharmaceuticals. ACS Omega.

[10] Ogawa Y., Tokunaga E., Kobayashi O., Hirai K., Shibata N. (2020). Current Contributions of Organofluorine Compounds to the Agrochemical Industry. iScience.

[11] Buer B.C., Marsh E.N. (2012). Fluorine: A new element in protein design. Protein Sci..

[12] Park B.K., Kitteringham N.R., Neill P.M. (2001). Metabolism of Fluorine-Containing Drugs. Annu. Rev. Pharmacol. Toxicol..

[13] Yamazaki T., Taguchi T., Ojima I. (2009). Unique Properties of Fluorine and their Relevance to Medicinal Chemistry and Chemical Biology. Fluorine in Medicinal Chemistry and Chemical Biology.

[14] Shah P., Westwell A.D. (2007). The role of fluorine in medicinal chemistry. J. Enzym. Inhib. Med. Chem..

[15] Hunter L. (2010). The C-F bond as a conformational tool in organic and biological chemistry. Beilstein J. Org. Chem..

[16] Muller K., Faeh C., Diederich F. (2007). Fluorine in pharmaceuticals: Looking beyond intuition. Science.

[17] Meanwell N.A. (2015). The Influence of Bioisosteres in Drug Design: Tactical Applications to Address Developability Problems. Tactics Contemp. Drug Des..

[18] Meanwell N.A. (2018). Fluorine and Fluorinated Motifs in the Design and Application of Bioisosteres for Drug Design. J. Med. Chem..

[19] Liu P., Sharon A., Chu C.K. (2008). Fluorinated nucleosides: Synthesis and biological implication. J. Fluor. Chem..

[20] Evich M., Spring-Connell A.M., Germann M.W. (2017). Impact of modified ribose sugars on nucleic acid conformation and function. Heterocycl. Commun..

[21] Cavaliere A., Probst K.C., Westwell A.D., Slusarczyk M. (2017). Fluorinated nucleosides as an important class of anticancer and antiviral agents. Future Med. Chem..

[22] Maderia M., Shenoy S., Van Q.N., Marquez V.E., Barchi J.J. (2007). Biophysical studies of DNA modified with conformationally constrained nucleotides: Comparison of 2′-exo (north) and 3′-exo (south) ‘locked’ templates. Nucleic Acids Res..

[23] Taylor A.I., Houlihan G., Holliger P. (2019). Beyond DNA and RNA: The Expanding Toolbox of Synthetic Genetics. Cold Spring Harb. Perspect. Biol..

[24] Schaerer O.D., Verdine G.L. (1995). A Designed Inhibitor of Base-Excision DNA Repair. J. Am. Chem. Soc..

[25] Lee S., Bowman B.R., Ueno Y., Wang S., Verdine G.L. (2008). Synthesis and structure of duplex DNA containing the genotoxic nucleobase lesion N7-methylguanine. J. Am. Chem. Soc..

[26] Codington J.F., Doerr I.L., Fox J.J. (1964). Nucleosides. XVIII. Synthesis of 2′-Fluorothymidine, 2′-Fluorodeoxyuridine, and Other 2′-Halogeno-2′-Deoxy Nucleosides1,2. J. Org. Chem..

[27] Hevey R. (2021). The Role of Fluorine in Glycomimetic Drug Design. Chem. Eur. J..

[28] Fukushima M., Fujioka A., Uchida J., Nakagawa F., Takechi T. (2001). Thymidylate synthase (TS) and ribonucleotide reductase (RNR) may be involved in acquired resistance to 5-fluorouracil (5-FU) in human cancer xenografts in vivo. Eur. J. Can..

[29] Shet H., Sahu R., Sanghvi Y.S., Kapdi A.R. (2022). Strategies for the Synthesis of Fluorinated Nucleosides, Nucleotides and Oligonucleotides. Chem. Rec..

[30] Pal S., Chandra G., Patel S., Singh S. (2022). Fluorinated Nucleosides: Synthesis, Modulation in Conformation and Therapeutic Application. Chem. Rec..

[31] Choi Y.K. (2021). Emerging and re-emerging fatal viral diseases. Exp. Mol. Med..

[32] Jordheim L.P., Durantel D., Zoulim F., Dumontet C. (2013). Advances in the development of nucleoside and nucleotide analogues for cancer and viral diseases. Nat. Rev. Drug Dis..

[33] Holec A.D., Mandal S., Prathipati P.K., Destache C.J. (2017). Nucleotide Reverse Transcriptase Inhibitors: A Thorough Review, Present Status and Future Perspective as HIV Therapeutics. Curr. Hiv. Res..

[34] Ismail M.M.F., Ayoup M.S. (2022). Review on fluorinated nucleoside/non-nucleoside FDA-approved antiviral drugs. Rsc. Adv..

[35] Roy V., Agrofoglio L.A. (2022). Nucleosides and emerging viruses: A new story. Drug Discov. Today.

[36] Carmine A.A., Brogden R.N., Heel R.C., Speight T.M., Avery G.S. (1982). Trifluridine: A review of its antiviral activity and therapeutic use in the topical treatment of viral eye infections. Drugs.

[37] Kaufman H.E., Heidelberger C. (1964). Therapeutic Antiviral Action of 5-Trifluoromethyl-2′-Deoxyuridine in Herpes Simplex Keratitis. Science.

[38] Umeda M., Heidelberger C. (1968). Comparative studies of fluorinated pyrimidines with various cell lines. Cancer Res..

[39] Yamashita J., Takeda S., Matsumoto H., Unemi N., Yasumoto M. (1989). Studies on antitumor agents. 8. Antitumor activities of O-alkyl derivatives of 2′-deoxy-5-(trifluoromethyl)uridine and 2′-deoxy-5-fluorouridine. J. Med. Chem..

[40] Prusoff W.H., Zucker M., Mancini W.R., Otto M.J., Lin T.S., Lee J.J. (1985). Basic biochemical and pharmacological aspects of antiviral agents. Antivir. Res..

[41] Wilhelmus K.R. (2015). Antiviral treatment and other therapeutic interventions for herpes simplex virus epithelial keratitis. Cochrane Database Syst. Rev..

[42] Matsuoka K., Nakagawa F., Kobunai T., Takechi T. (2018). Trifluridine/tipiracil overcomes the resistance of human gastric 5-fluorouracil-refractory cells with high thymidylate synthase expression. Oncotarget.

[43] Burness C.B., Duggan S.T. (2016). Trifluridine/Tipiracil: A Review in Metastatic Colorectal Cancer. Drugs.

[44] Andersen S.E., Andersen I.B., Jensen B.V., Pfeiffer P., Ota T., Larsen J.S. (2019). A systematic review of observational studies of trifluridine/tipiracil (TAS-102) for metastatic colorectal cancer. Acta. Oncol..

[45] Heidelberger C., Parsons D., Remy D.C. (1962). Syntheses of 5-trifluoromethyluracil and 5-trifluoromethyl-2 ″-deoxyuridine. J. Am. Chem. Soc..

[46] Kobayashi Y., Yamamoto K., Asai T., Nakano M., Kumadaki I. (1980). Studies on organic fluorine compounds. Part 35. Trifluoromethylation of pyrimidine- and purine-nucleosides with trifluoromethyl–copper complex. J. Chem. Soc. Perkin Trans..

[47] Kawakami H., Takashi E., Koshi K., Hajime M., Yoshitake N., Kazuo I., Nobuhiro M. (1990). The Synthesis of 2′-Deoxy-5-trifluoromethyluridine Utilizing a Coupling Reaction. Heterocycles.

[48] Komatsu H., Umetani H. (2002). Synthesis of Trifluorothymidine:  Green Glycosylation Condition Using Neither Chloroform nor Transition Metals. Org. Process Res. Dev..

[49] Salvetti R., Pregnolato M., Verri A., Focher F., Spadari S., Marchand A., Mathe C., Gosselin G. (2001). Synthesis and In Vitro Activity of D- and L-Enantiomers of 5-(Trifluoromethyl)Uracil Nucleoside Derivatives. Nucleosides Nucleotides Nucleic Acids.

[50] Kataoka Y., Iimori M., Niimi S., Tsukihara H., Wakasa T., Saeki H., Oki E., Maehara Y., Kitao H. (2019). Cytotoxicity of trifluridine correlates with the thymidine kinase 1 expression level. Sci. Rep..

[51] Safrin S. (1996). Treatment of acyclovir-resistant herpes simplex and varicella zoster virus infections. Antiv. Chemother. 4.

[52] Frampton J.E., Perry C.M. (2005). Emtricitabine—A review of its use in the management of HIV infection. Drugs.

[53] Liotta D.C., Painter G.R. (2016). Discovery and Development of the Anti-Human Immunodeficiency Virus Drug, Emtricitabine (Emtriva, FTC). Acc. Chem. Res..

[54] Beach J.W., Jeong L.S., Alves A.J., Pohl D., Kim H.O., Chang C.N., Doong S.L., Schinazi R.F., Cheng Y.C., Chu C.K. (1992). Synthesis of enantiomerically pure (2′R,5′S)-(-)-1-(2-hydroxymethyloxathiolan-5-yl)cytosine as a potent antiviral agent against hepatitis B virus (HBV) and human immunodeficiency virus (HIV). J. Org. Chem..

[55] Schinazi R.F., McMillan A., Cannon D., Mathis R., Lloyd R.M., Peck A., Sommadossi J.P., St Clair M., Wilson J., Furman P.A. (1992). Selective inhibition of human immunodeficiency viruses by racemates and enantiomers of cis-5-fluoro-1-[2-(hydroxymethyl)-1,3-oxathiolan-5-yl]cytosine. Antimicrob. Agents Chemother..

[56] Schinazi R.F., Boudinot F.D., Ibrahim S.S., Manning C., McClure H.M., Liotta D.C. (1992). Pharmacokinetics and metabolism of racemic 2′,3′-dideoxy-5-fluoro-3′-thiacytidine in rhesus monkeys. Antimicrob. Agents Chemother..

[57] Mandala D., Thompson W.A., Watts P. (2016). Synthesis routes to anti-HIV drugs. Tetrahedron.

[58] Gumina G., Song G.Y., Chu C.K. (2001). L-Nucleosides as chemotherapeutic agents. FEMS Microbiol. Lett..

[59] Kraus J.L., Attardo G. (1991). Synthesis of New 2,5-Substituted 1,3-Oxathiolanes. Intermediates in Nucleoside Chemistry. Synthesis.

[60] Jin H., Siddiqui M.A., Evans C.A., Tse H.A., Mansour T.S., Goodyear M.D., Ravenscroft P., Beels C.D. (1995). Diastereoselective synthesis of the potent antiviral agent (-)-2′-deoxy-3′-thiacytidine and its enantiomer. J. Org. Chem..

[61] Cousins R.P.C., Mahmoudian M., Youds P.M. (1995). Enzymic resolution of oxathiolane intermediates—An alternative approach to the anti-viral agent lamivudine (3TC™). Tetrahedron Asymmetry.

[62] Kraus J.L. (1993). New Phosphonate Analogues of 3′-Thia-2′,3′-dideoxycytidine(BCH-189) Synthesis and Anti-HIV Evaluation. Nucleosides Nucleotides.

[63] Snead D.R., McQuade D.T., Ahmad S., Krack R., Stringham R.W., Burns J.M., Abdiaj I., Gopalsamuthiram V., Nelson R.C., Gupton B.F. (2020). An Economical Route to Lamivudine Featuring a Novel Strategy for Stereospecific Assembly. Org. Process Rese. Dev..

[64] Goodyear M.D., Hill M.L., West J.P., Whitehead A.J. (2005). Practical enantioselective synthesis of lamivudine (3TC (TM)) via a dynamic kinetic resolution. Tetrahedron Lett..

[65] Mandala D., Watts P. (2017). An Improved Synthesis of Lamivudine and Emtricitabine. Chemistryselect.

[66] Kashinath K., Snead D.R., Burns J.M., Stringham R.W., Gupton B.F., McQuade D.T. (2020). Synthesis of an Oxathiolane Drug Substance Intermediate Guided by Constraint-Driven Innovation. Org. Process Res. Dev..

[67] Emtriva (Emtricitabine) Capsule Label. https://www.accessdata.fda.gov/drugsatfda_docs/label/2012/021500s019lbl.pdf.

[68] Ng H.H., Stock H., Rausch L., Bunin D., Wang A., Brill S., Gow J., Mirsalis J.C. (2015). Tenofovir disoproxil fumarate: Toxicity, toxicokinetics, and toxicogenomics analysis after 13 weeks of oral administration in mice. Int. J. Toxicol..

[69] Richman D.D. (2001). Antiretroviral activity of emtricitabine, a potent nucleoside reverse transcriptase inhibitor. Antivir. Ther..

[70] Perry C.M. (2014). Elvitegravir/Cobicistat/Emtricitabine/Tenofovir Disoproxil Fumarate Single-Tablet Regimen (Stribild^®^): A Review of Its Use in the Management of HIV-1 Infection in Adults. Drugs.

[71] Sofia M.J., Bao D., Chang W., Du J., Nagarathnam D., Rachakonda S., Reddy P.G., Ross B.S., Wang P., Zhang H.R. (2010). Discovery of a beta-d-2′-deoxy-2′-alpha-fluoro-2′-beta-C-methyluridine nucleotide prodrug (PSI-7977) for the treatment of hepatitis C virus. J. Med. Chem..

[72] Khalil A., El-Khouly A.S., Elkaeed E.B., Eissa I.H. (2022). The Inhibitory Potential of 2′-dihalo Ribonucleotides against HCV: Molecular Docking, Molecular Simulations, MM-BPSA, and DFT Studies. Molecules.

[73] Appleby T.C., Perry J.K., Murakami E., Barauskas O., Feng J., Cho A., Fox D., Wetmore D.R., McGrath M.E., Ray A.S. (2015). Structural basis for RNA replication by the hepatitis C virus polymerase. Science.

[74] de Albuquerque P., Santos L.H.S., Antunes D., Caffarena E.R., Figueiredo A.S. (2020). Structural insights into NS5B protein of novel equine hepaciviruses and pegiviruses complexed with polymerase inhibitors. Virus Res..

[75] Murakami E., Niu C., Bao H., Micolochick Steuer H.M., Whitaker T., Nachman T., Sofia M.A., Wang P., Otto M.J., Furman P.A. (2008). The mechanism of action of beta-D-2′-deoxy-2′-fluoro-2′-C-methylcytidine involves a second metabolic pathway leading to beta-D-2′-deoxy-2′-fluoro-2′-C-methyluridine 5′-triphosphate, a potent inhibitor of the hepatitis C virus RNA-dependent RNA polymerase. Antimicrob. Agents Chemother..

[76] FDA Approves New Treatment for Pediatric Patients with Any Strain of Hepatitis C. https://www.fda.gov/news-events/press-announcements/fda-approves-new-treatment-pediatric-patients-any-strain-hepatitis-c.

[77] Xiao F., Fofana I., Thumann C., Mailly L., Alles R., Robinet E., Meyer N., Schaeffer M., Habersetzer F., Doffoel M. (2015). Synergy of entry inhibitors with direct-acting antivirals uncovers novel combinations for prevention and treatment of hepatitis C. Gut.

[78] Clark J.L., Hollecker L., Mason J.C., Stuyver L.J., Tharnish P.M., Lostia S., McBrayer T.R., Schinazi R.F., Watanabe K.A., Otto M.J. (2005). Design, synthesis, and antiviral activity of 2′-deoxy-2′-fluoro-2′-C-methylcytidine, a potent inhibitor of hepatitis C virus replication. J. Med. Chem..

[79] Pockros P.J. (2008). Emerging therapies for chronic hepatitis C virus. Gastroenterol. Hepatol..

[80] Wang P., Chun B.K., Rachakonda S., Du J., Khan N., Shi J., Stec W., Cleary D., Ross B.S., Sofia M.J. (2009). An efficient and diastereoselective synthesis of PSI-6130: A clinically efficacious inhibitor of HCV NS5B polymerase. J. Org. Chem..

[81] Gao Y., Sharpless K.B. (1988). Vicinal diol cyclic sulfates. Like epoxides only more reactive. J. Am. Chem. Soc..

[82] Ma H., Jiang W.R., Robledo N., Leveque V., Ali S., Lara-Jaime T., Masjedizadeh M., Smith D.B., Cammack N., Klumpp K. (2007). Characterization of the metabolic activation of hepatitis C virus nucleoside inhibitor beta-D-2′-Deoxy-2′-fluoro-2′-C-methylcytidine (PSI-6130) and identification of a novel active 5′-triphosphate species. J. Biol. Chem..

[83] Murakami E., Bao H., Ramesh M., McBrayer T.R., Whitaker T., Micolochick Steuer H.M., Schinazi R.F., Stuyver L.J., Obikhod A., Otto M.J. (2007). Mechanism of activation of beta-D-2′-deoxy-2′-fluoro-2′-c-methylcytidine and inhibition of hepatitis C virus NS5B RNA polymerase. Antimicrob. Agents Chemother..

[84] Thornton P.J., Kadri H., Miccoli A., Mehellou Y. (2016). Nucleoside Phosphate and Phosphonate Prodrug Clinical Candidates. J. Med. Chem..

[85] Slusarczyk M., Serpi M., Pertusati F. (2018). Phosphoramidates and phosphonamidates (ProTides) with antiviral activity. Antivir. Chem. Chemother..

[86] Ross B.S., Reddy P.G., Zhang H.R., Rachakonda S., Sofia M.J. (2011). Synthesis of Diastereomerically Pure Nucleotide Phosphoramidates. J. Org. Chem..

[87] EPCLUSA^®^ (Sofosbuvir and Velpatasvir) Tablets, for Oral Use Initial U.S. Approval: 2016. https://www.accessdata.fda.gov/drugsatfda_docs/label/2017/208341s009lbl.pdf.

[88] Sulkowski M.S. (2014). Daclatasvir plus Sofosbuvir for Previously Treated or Untreated Chronic HCV Infection. N. Engl. J. Med..

[89] Mumtaz N., Jimmerson L.C., Bushman L.R., Kiser J.J., Aron G., Reusken C., Koopmans M.P.G., van Kampen J.J.A. (2017). Cell-line dependent antiviral activity of sofosbuvir against Zika virus. Antivir. Res..

[90] Dragoni F., Boccuto A., Picarazzi F., Giannini A., Giammarino F., Saladini F., Mori M., Mastrangelo E., Zazzi M., Vicenti I. (2020). Evaluation of sofosbuvir activity and resistance profile against West Nile virus in vitro. Antivir. Res..

[91] Xu H.T., Colby-Germinario S.P., Hassounah S.A., Fogarty C., Osman N., Palanisamy N., Han Y.S., Oliveira M., Quan Y.D., Wainberg M.A. (2017). Evaluation of Sofosbuvir (beta-D-2′-deoxy-2′-alpha-fluoro-2′-beta-C-methyluridine) as an inhibitor of Dengue virus replication. Sci. Rep..

[92] Chu C.K., Ma T., Shanmuganathan K., Wang C., Xiang Y., Pai S.B., Yao G.Q., Sommadossi J.P., Cheng Y.C. (1995). Use of 2′-fluoro-5-methyl-beta-L-arabinofuranosyluracil as a novel antiviral agent for hepatitis B virus and Epstein-Barr virus. Antimicrob. Agents Chemother..

[93] Sharon A., Jha A.K., Chu C.K. (2010). Clevudine, to Treat Hepatitis B Viral Infection. Analogue-Based Drug Discovery II; edn..

[94] Du J., Choi Y., Lee K., Chun B.K., Hong J.H., Chu C.K. (1999). A practical synthesis of L-FMAU from L-arabinose. Nucleosides Nucleotides.

[95] Sznaidman M.L., Almond M.R., Pesyan A. (2002). New Synthesis of L-Fmau from L-Arabinose. Nucleosides Nucleotides Nucleic Acids.

[96] Tremblay T., Alcée J.B., Giguère D. (2022). Protecting-group-free synthesis of clevudine (l-FMAU), a treatment of the hepatitis B virus. Org. Biomol. Chem..

[97] Yao G.Q., Liu S.H., Chou E., Kukhanova M., Chu C.K., Cheng Y.C. (1996). Inhibition of Epstein-Barr virus replication by a novel L-nucleoside, 2′-fluoro-5-methyl-beta-L-arabinofuranosyluracil. Biochem. Pharmacol..

[98] Niu C.R., Bao H.Y., Tolstykh T., Steuer H.M.M., Murakami E., Korba B., Furman P.A. (2010). Evaluation of the in vitro anti-HBV activity of clevudine in combination with other nucleoside/nucleotide inhibitors. Antivir. Ther..

[99] Lee H.S., Chung Y.H., Lee K., Byun K.S., Paik S.W., Han J.Y., Yoo K., Yoo H.W., Lee J.H., Yoo B.C. (2006). A 12-week clevudine therapy showed potent and durable antiviral activity in HBeAg-positive chronic hepatitis B. Hepatology.

[100] Korba B.E., Furman P.A., Otto M.J. (2006). Clevudine: A potent inhibitor of hepatitis B virus in vitro and in vivo. Expert Rev. Anti. Infect. Ther..

[101] Peek S.F., Cote P.J., Jacob J.R., Toshkov I.A., Hornbuckle W.E., Baldwin B.H., Wells F.V., Chu C.K., Gerin J.L., Tennant B.C. (2001). Antiviral activity of clevudine [L-FMAU, (1-(2-fluoro-5-methyl-beta, L-arabinofuranosyl) uracil)] against woodchuck hepatitis virus replication and gene expression in chronically infected woodchucks (Marmota monax). Hepatology.

[102] An Open-Label Treatment Protocol to Provide Continued Elvucitabine Treatment. https://classic.clinicaltrials.gov/ct2/show/NCT00675844.

[103] Esposito F., Corona A., Tramontano E. (2012). HIV-1 Reverse Transcriptase Still Remains a New Drug Target: Structure, Function, Classical Inhibitors, and New Inhibitors with Innovative Mechanisms of Actions. Mol. Biol. Int..

[104] Lin T.S., Luo M.Z., Liu M.C., Zhu Y.L., Gullen E., Dutschman G.E., Cheng Y.C. (1996). Design and synthesis of 2′,3′-dideoxy-2′,3′-didehydro-beta-L-cytidine (beta-L-d4C) and 2′,3′-dideoxy 2′,3′-didehydro-beta-L-5-fluorocytidine (beta-L-Fd4C), two exceptionally potent inhibitors of human hepatitis B virus (HBV) and potent inhibitors of human immunodeficiency virus (HIV) in vitro. J. Med. Chem..

[105] Chen S.H., Li X.Y., Li J., Niu C.S., Carmichael E., Doyle T.W. (1997). Stereoselective syntheses of beta-L-FD4C and beta-L-FddC. J. Org. Chem..

[106] Kim K.H., Kim N.D., Seong B.L. (2010). Discovery and Development of Anti-HBV Agents and Their Resistance. Molecules.

[107] Colucci P., Pottage J.C., Robison H., Turgeon J., Schurmann D., Hoepelman I.M., Ducharme M.P. (2009). Multiple-Dose Pharmacokinetic Behavior of Elvucitabine, a Nucleoside Reverse Transcriptase Inhibitor, Administered over 21 Days with Lopinavir-Ritonavir in Human Immunodeficiency Virus Type 1-Infected Subjects. Antimicrob. Agents Chemother..

[108] Chen S.H., Wang Q., Mao J., King I., Dutschman G.E., Gullen E.A., Cheng Y.C., Doyle T.W. (1998). Synthesis and biological evaluation of a series of 2′-fluorinated-2′,3′-dideoxy-2′,3′-didehydro-(l)-nucleosides. Bioorg. Med. Chem. Lett..

[109] Shi J., McAtee J.J., Schlueter Wirtz S., Tharnish P., Juodawlkis A., Liotta D.C., Schinazi R.F. (1999). Synthesis and Biological Evaluation of 2‘,3‘-Didehydro-2‘,3‘-dideoxy-5- fluorocytidine (D4FC) Analogues:  Discovery of Carbocyclic Nucleoside Triphosphates with Potent Inhibitory Activity against HIV-1 Reverse Transcriptase. J. Med. Chem..

[110] The Safety and Effectiveness of FIAC in the Treatment of Cytomegalovirus (CMV) in Patients with AIDS. https://clinicaltrials.gov/ct2/show/NCT00000981?term=Fiacitabine&draw=2&rank=1.

[111] Watanabe K.A., Su T.L., Klein R.S., Chu C.K., Matsuda A., Chun M.W., Lopez C., Fox J.J. (1983). Nucleosides. 123. Synthesis of antiviral nucleosides: 5-substituted 1-(2-deoxy-2-halogeno-beta-D-arabinofuranosyl)cytosines and -uracils. Some structure-activity relationships. J. Med. Chem..

[112] Rollinson E.A. (1987). Comparative efficacy of three 2′-fluoropyrimidine nucleosides and 9-(1,3-dihydroxy-2-propoxymethyl)guanine (BW B759U) against pseudorabies and equine rhinopneumonitis virus infection in vitro and in laboratory animals. Antivir. Res..

[113] Coen N., Duraffour S., Topalis D., Snoeck R., Andrei G. (2014). Spectrum of activity and mechanisms of resistance of various nucleoside derivatives against gammaherpesviruses. Antimicrob. Agents Chemother..

[114] Colacino J.M., Lopez C. (1983). Efficacy and Selectivity of Some Nucleoside Analogs as Anti-Human Cytomegalovirus Agents. Antimicrob. Agents Chemother..

[115] Nikkels A.F., Pierard G.E. (1994). Recognition and treatment of shingles. Drugs.

[116] Molina J.M., Yazdanpanah Y., Afani Saud A., Bettacchi C., Chahin Anania C., Klopfer S.O., Grandhi A., Eves K., Hepler D., Robertson M.N. (2022). Brief Report: Efficacy and Safety of Oral Islatravir Once Daily in Combination with Doravirine through 96 Weeks for Treatment-Naive Adults with HIV-1 Infection Receiving Initial Treatment with Islatravir, Doravirine, and Lamivudine. J. Acquir. Immune. Defic. Syndr..

[117] Markowitz M., Sarafianos S.G. (2018). 4′-Ethynyl-2-fluoro-2′-deoxyadenosine, MK-8591: A novel HIV-1 reverse transcriptase translocation inhibitor. Curr. Opin. HIV AIDS.

[118] MK-8591. https://clinicaltrials.gov/ct2/results?cond=&term=mk-8591&cntry=&state=&city=&dist=.

[119] Kohgo S., Yamada K., Kitano K., Iwai Y., Sakata S., Ashida N., Hayakawa H., Nameki D., Kodama E., Matsuoka M. (2004). Design, efficient synthesis, and anti-HIV activity of 4′-C-cyano- and 4′-C-ethynyl-2′-deoxy purine nucleosides. Nucleosides Nucleotides Nucleic Acids.

[120] Ohrui H., Kohgo S., Hayakawa H., Kodama E., Matsuoka M., Nakata T., Mitsuya H. (2006). 2′-Deoxy-4′-C-ethynyl-2-fluoroadenosine: A nucleoside reverse transcriptase inhibitor with highly potent activity against all HIV-1 strains, favorable toxic profiles and stability in plasma. Nucleic Acids Symp. Ser..

[121] Schurmann D., Rudd D.J., Zhang S., De Lepeleire I., Robberechts M., Friedman E., Keicher C., Huser A., Hofmann J., Grobler J.A. (2020). Safety, pharmacokinetics, and antiretroviral activity of islatravir (ISL, MK-8591), a novel nucleoside reverse transcriptase translocation inhibitor, following single-dose administration to treatment-naive adults infected with HIV-1: An open-label, phase 1b, consecutive-panel trial. Lancet HIV.

[122] Salie Z.L., Kirby K.A., Michailidis E., Marchand B., Singh K., Rohan L.C., Kodama E.N., Mitsuya H., Parniak M.A., Sarafianos S.G. (2016). Structural basis of HIV inhibition by translocation-defective RT inhibitor 4′-ethynyl-2-fluoro-2′-deoxyadenosine (EFdA). Proc. Natl. Acad. Sci. USA.

[123] Ohrui H. (2006). 2′-deoxy-4′-C-ethynyl-2-fluoroadenosine, a nucleoside reverse transcriptase inhibitor, is highly potent against all human immunodeficiency viruses type 1 and has low toxicity. Chem. Rec..

[124] Kageyama M., Miyagi T., Yoshida M., Nagasawa T., Ohrui H., Kuwahara S. (2012). Concise synthesis of the anti-HIV nucleoside EFdA. Biosci. Biotechnol. Biochem..

[125] Fukuyama K., Ohrui H., Kuwahara S. (2015). Synthesis of EFdA via a diastereoselective aldol reaction of a protected 3-keto furanose. Org. Lett..

[126] McLaughlin M., Kong J., Belyk K.M., Chen B., Gibson A.W., Keen S.P., Lieberman D.R., Milczek E.M., Moore J.C., Murray D. (2017). Enantioselective Synthesis of 4′-Ethynyl-2-fluoro-2′-deoxyadenosine (EFdA) via Enzymatic Desymmetrization. Org. Lett..

[127] Patel N.R., Nawrat C.C., McLaughlin M., Xu Y., Huffman M.A., Yang H., Li H., Whittaker A.M., Andreani T., Lévesque F. (2020). Synthesis of Islatravir Enabled by a Catalytic, Enantioselective Alkynylation of a Ketone. Org. Lett..

[128] Huffman M.A., Fryszkowska A., Alvizo O., Borra-Garske M., Campos K.R., Canada K.A., Devine P.N., Duan D., Forstater J.H., Grosser S.T. (2019). Design of an in vitro biocatalytic cascade for the manufacture of islatravir. Science.

[129] Stoddart C.A., Galkina S.A., Joshi P., Kosikova G., Moreno M.E., Rivera J.M., Sloan B., Reeve A.B., Sarafianos S.G., Murphey-Corb M. (2015). Oral Administration of the Nucleoside EFdA (4′-Ethynyl-2-Fluoro-2′-Deoxyadenosine) Provides Rapid Suppression of HIV Viremia in Humanized Mice and Favorable Pharmacokinetic Properties in Mice and the Rhesus Macaque. Antimicrob. Agents Chemother..

[130] Chen Y.C., Haznedar J., Kulkarni R., Vistuer C., Washington C., Liu M., Smith P. (2014). Evaluation of the effect of mericitabine at projected therapeutic and supratherapeutic doses on cardiac repolarization in healthy subjects: A thorough QT/QTc study. Clin. Pharmacol. Drug Dev..

[131] Stuyver L.J., McBrayer T.R., Tharnish P.M., Clark J., Hollecker L., Lostia S., Nachman T., Grier J., Bennett M.A., Xie M.Y. (2006). Inhibition of hepatitis C replicon RNA synthesis by β-D-2′-deoxy-2′-fluoro-2′-C-methylcytidine: A specific inhibitor of hepatitis C virus replication. Antivir. Chem. Chemother..

[132] Washington C., Moreira S., Haznedar J., Goelzer P., Chen Y.C. (2014). Single-dose Pharmacokinetics of the HCV Polymerase Inhibitor Mericitabine in Healthy Caucasian and Japanese Subjects. Drug Metab. Pharmacokinet..

[133] Mericitabine. https://clinicaltrials.gov/ct2/results?cond=&term=Mericitabine&cntry=&state=&city=&dist=.

[134] Carr R., Hildbrand S., Hodges M.L., Kammrer M., Lang J.F., Lawrimore W.J., Nguyen D. (2013). Process for the Preparation of 2-Deoxy-2-fluoro-2-methyl-D-dibofuranosyl Nucleoside Compounds. U.S. Patent.

[135] Pawlotsky J.M., Najera I., Jacobson I. (2012). Resistance to mericitabine, a nucleoside analogue inhibitor of HCV RNA-dependent RNA polymerase. Antivir. Ther..

[136] Ali S., Leveque V., Le Pogam S., Ma H., Philipp F., Inocencio N., Smith M., Alker A., Kang H., Najera I. (2008). Selected Replicon Variants with Low-Level In Vitro Resistance to the Hepatitis C Virus NS5B Polymerase Inhibitor PSI-6130 Lack Cross-Resistance with R1479. Antimicrob. Agents Chemother..

[137] Reddy P.G., Bao D., Chang W., Chun B.K., Du J., Nagarathnam D., Rachakonda S., Ross B.S., Zhang H.R., Bansal S. (2010). 2′-deoxy-2′-alpha-fluoro-2′-beta-C-methyl 3′,5′-cyclic phosphate nucleotide prodrug analogs as inhibitors of HCV NS5B polymerase: Discovery of PSI-352938. Bioorg. Med. Chem. Lett..

[138] Pockros P.J., Jensen D., Tsai N., Taylor R., Ramji A., Cooper C., Dickson R., Tice A., Kulkarni R., Vierling J.M. (2013). A randomized trial of mericitabine plus pegylated interferon alpha-2a/ribavirin for 24 weeks in treatment-naive HCV genotype 1/4 patients. Hepatology.

[139] Good S.S., Moussa A., Zhou X.J., Pietropaolo K., Sommadossi J.P. (2020). Preclinical evaluation of AT-527, a novel guanosine nucleotide prodrug with potent, pan-genotypic activity against hepatitis C virus. PLoS ONE.

[140] Good S.S., Westover J., Jung K.H., Zhou X.J., Moussa A., La Colla P., Collu G., Canard B., Sommadossi J.P. (2021). AT-527, a Double Prodrug of a Guanosine Nucleotide Analog, Is a Potent Inhibitor of SARS-CoV-2 In Vitro and a Promising Oral Antiviral for Treatment of COVID-19. Antimicrob. Agents Chemother..

[141] Berliba E., Bogus M., Vanhoutte F., Berghmans P.J., Good S.S., Moussa A., Pietropaolo K., Murphy R.L., Zhou X.J., Sommadossi J.P. (2019). Safety, pharmacokinetics and antiviral activity of AT-527, a novel purine nucleotide prodrug, in HCV-infected subjects with and without cirrhosis. Antimicrob. Agents Chemother..

[142] https://clinicaltrials.gov/study/NCT05904470.

[143] Moussa A. (2022). Stereoselective Manufacture of Selected Purine Phosphoramidates. U.S. Patent.

[144] Study to Evaluate the Effects of AT-527 in Non-Hospitalized Adult Patients with Mild or Moderate COVID-19. https://classic.clinicaltrials.gov/ct2/show/NCT04709835.

[145] AT-752. https://clinicaltrials.gov/ct2/results?cond=&term=AT-752&cntry=&state=&city=&dist=.

[146] Good S.S., Shannon A., Lin K., Moussa A., Julander J.G., La Colla P., Collu G., Canard B., Sommadossi J.P. (2021). Evaluation of AT-752, a Double Prodrug of a Guanosine Nucleotide Analog with In Vitro and In Vivo Activity against Dengue and Other Flaviviruses. Antimicrob. Agents Chemother..

[147] Study of AT-752 in Healthy Subjects. https://clinicaltrials.gov/study/NCT04722627?cond=AT-752&rank=1.

[148] Lin K., Good S.S., Julander J.G., Weight A.E., Moussa A., Sommadossi J.P. (2022). AT-752, a double prodrug of a guanosine nucleotide analog, inhibits yellow fever virus in a hamster model. PLoS Negl. Trop. Dis..

[149] https://clinicaltrials.gov/study/NCT02339207?cond=HCV%20Infection&term=AL-335&rank=2.

[150] https://clinicaltrials.gov/study/NCT02569710?cond=HCV%20Infection&term=AL-335&rank=3&tab=results.

[151] Wang G., Dyatkina N., Prhavc M., Williams C., Serebryany V., Hu Y., Huang Y., Wan J., Wu X., Deval J. (2019). Synthesis and Anti-HCV Activities of 4′-Fluoro-2′-Substituted Uridine Triphosphates and Nucleotide Prodrugs: Discovery of 4′-Fluoro-2′-C-methyluridine 5′-Phosphoramidate Prodrug (AL-335) for the Treatment of Hepatitis C Infection. J. Med. Chem..

[152] McClure M.W., Berliba E., Tsertsvadze T., Streinu-Cercel A., Vijgen L., Astruc B., Patat A., Westland C., Chanda S., Zhang Q. (2018). Safety, tolerability, and pharmacokinetics of AL-335 in healthy volunteers and hepatitis C virus-infected subjects. PLoS ONE.

[153] Leonid B., Guangyi W., David B.S. (2016). Substituted Nucleosides, Nucleotdes and Analogs Thereof. U.S. Patent.

[154] Wang G., Lim S.P., Chen Y.L., Hunziker J., Rao R., Gu F., She C.C., Ghafar N.A., Xu H.Y., Chan K. (2018). Structure-activity relationship of uridine-based nucleoside phosphoramidate prodrugs for inhibition of dengue virus RNA-dependent RNA polymerase. Bioorg. Med. Chem. Lett..

[155] Singh U.S., Mulamoottil V.A., Chu C.K. (2018). 2′-Fluoro-6′-methylene carbocyclic adenosine and its phosphoramidate prodrug: A novel anti-HBV agent, active against drug-resistant HBV mutants. Med. Res. Rev..

[156] Rawal R.K., Singh U.S., Chavre S.N., Wang J.N., Sugiyama M., Hung W., Govindarajan R., Korba B., Tanaka Y., Chu C.K. (2013). 2 ‘-Fluoro-6 ‘-methylene-carbocyclic adenosine phosphoramidate (FMCAP) prodrug: In vitro anti-HBV activity against the lamivudine-entecavir resistant triple mutant and its mechanism of action. Bioorg. Med. Chem. Lett..

[157] Gadthula S., Rawal R.K., Sharon A., Wu D., Korba B., Chu C.K. (2011). Synthesis and antiviral activity of cyclopropyl-spirocarbocyclic adenosine, (4R,5S,6R,7R)-4-(6-amino-9H-purin-9-yl)-7-(hydroxymethyl)spiro[2.4]heptane-5,6-diol against hepatitis C virus. Bioorg. Med. Chem. Lett..

[158] Jin Y.H., Liu P., Wang J., Baker R., Huggins J., Chu C.K. (2003). Practical Synthesis of d- and l-2-Cyclopentenone and Their Utility for the Synthesis of Carbocyclic Antiviral Nucleosides against Orthopox Viruses (Smallpox, Monkeypox, and Cowpox Virus). J. Org. Chem..

[159] Gemal A.L., Luche J.L. (1981). Lanthanoids in organic synthesis. 6. Reduction of. alpha.-enones by sodium borohydride in the presence of lanthanoid chlorides: Synthetic and mechanistic aspects. J. Am. Chem. Soc..

[160] Wang J.N., Singh U.S., Rawal R.K., Sugiyama M., Yoo J., Jha A.K., Scroggin M., Huang Z.H., Murray M.G., Govindarajan R. (2011). Antiviral activity of novel 2′-fluoro-6′-methylene-carbocyclic adenosine against wild-type and drug-resistant hepatitis B virus mutants. Bioorg. Med. Chem. Lett..

[161] Singh U.S., Mishra R.C., Shankar R., Chu C.K. (2014). Stereoselective Synthesis of 2′-Fluoro-6′-methylene Carbocyclic Adenosine via Vince Lactam. J. Org. Chem..

[162] Luche J.L. (1978). Lanthanides in Organic-Chemistry. 1. Selective 1,2 Reductions of Conjugated Ketones. J. Am. Chem. Soc..

[163] Singh U.S., Mulamoottil V.A., Chu C.K. (2019). Synthesis of an Anti-hepatitis B Agent, 2′-Fluoro-6′-methylene-carbocyclic Adenosine (FMCA) and Its Phosphoramidate (FMCAP). J. Org. Chem..

[164] Villet S., Ollivet A., Pichoud C., Barraud L., Villeneuve J.P., Trepo C., Zoulim F. (2007). Stepwise process for the development of entecavir resistance in a chronic hepatitis B virus infected patient. J. Hepatol..

[165] Walsh A.W., Langley D.R., Colonno R.J., Tenney D.J. (2010). Mechanistic Characterization and Molecular Modeling of Hepatitis B Virus Polymerase Resistance to Entecavir. PLoS ONE.

[166] Chang W., Bao D.H., Chun B.K., Naduthambi D., Nagarathnam D., Rachakonda S., Reddy P.G., Ross B.S., Zhang H.R., Bansal S. (2011). Discovery of PSI-353661, a Novel Purine Nucleotide Prodrug for the Treatment of HCV Infection. ACS Med. Chem. Lett..

[167] Karuna R., Yokokawa F., Wang K., Zhang J., Xu H., Wang G., Ding M., Chan W.L., Abdul Ghafar N., Leonardi A. (2020). A Cyclic Phosphoramidate Prodrug of 2′-Deoxy-2′-Fluoro-2′-C-Methylguanosine for the Treatment of Dengue Virus Infection. Antimicrob. Agents Chemother..

[168] Wang G., Dyatkina N., Prhavc M., Williams C., Serebryany V., Hu Y., Huang Y., Wu X., Chen T., Huang W. (2020). Synthesis and Anti-HCV Activity of Sugar-Modified Guanosine Analogues: Discovery of AL-611 as an HCV NS5B Polymerase Inhibitor for the Treatment of Chronic Hepatitis C. J. Med. Chem..

